# Clinical impact and cost-effectiveness of the WHO-recommended advanced HIV disease package of care

**DOI:** 10.1016/S2214-109X(25)00190-1

**Published:** 2025-07-22

**Authors:** Emily P Hyle, Thulani Maphosa, Ajay Rangaraj, Mary Feser, Geoffrey C Singini, Prakriti Shrestha, Amir Shroufi, Krishna P Reddy, Eddie Matiya, Rosalia Dambe, Virginia R Talbot, Rachel Chamanga, C Robert Horsburgh, Milton C Weinstein, Rose K Nyirenda, Nathan Ford, Appolinaire Tiam, Andrew Phillips, Kenneth A Freedberg

**Affiliations:** a Medical Practice Evaluation Center, Massachusetts General Hospital, Boston, MA, USA; b Division of Infectious Diseases, Massachusetts General Hospital, Boston, MA, USA; c Division of Pulmonary and Critical Care Medicine, Massachusetts General Hospital, Boston, MA, USA; d Division of General Internal Medicine, Massachusetts General Hospital, Boston, MA, USA; e Harvard Medical School, Boston, MA, USA; f Elizabeth Glaser Pediatric AIDS Foundation Malawi, Lilongwe, Malawi; g Department of Global HIV, Hepatitis, and Sexually Transmitted Diseases, World Health Organization, Geneva, Switzerland; h HIV Team, The Global Fund to Fight AIDS, Tuberculosis and Malaria, Geneva, Switzerland; i Department of Epidemiology, Biostatistics, Global Health and Medicine, Boston University, Boston, MA, USA; j Department of Health Policy and Management, Harvard T H Chan School of Public Health, Boston, MA, USA; k Division of HIV and AIDS, Ministry of Health, Government of Malawi, Lilongwe, Malawi; l Centre for Integrated Data and Epidemiological Research, School of Public Health and Family Medicine, University of Cape Town, Cape Town, South Africa; m Elizabeth Glaser Pediatric AIDS Foundation, Washington, DC, USA; n Department of Epidemiology, The George Washington University Milken Institute School of Public Health, Washington, DC, USA; o HIV Modelling Consortium, Institute for Global Health, University College London, London, UK

## Abstract

**Background:**

In sub-Saharan Africa, 20–40% of people living with HIV present with advanced HIV disease (AHD), which can be diagnosed, treated, and prevented using a package of care recommended by WHO. We aimed to project the cost-effectiveness and budget impact of the WHO-recommended AHD package in Malawi.

**Methods:**

Using the Cost-Effectiveness of Preventing AIDS Complications-International model, we simulated a cohort of non-hospitalised people living with HIV (aged >19 years) initiating antiretroviral therapy (ART), 25% of whom had AHD (CD4 count <200 cells per μL and/or WHO stage 3 or 4 disease). We assessed 13 increasingly comprehensive strategies, ranging from ART only to the WHO-recommended AHD package, including tuberculosis diagnostics (ie, sputum Xpert and urine lipoarabinomannan), tuberculosis preventive therapy, serum cryptococcal antigen (CrAg) screening with pre-emptive fluconazole treatment if CrAg-positive, and co-trimoxazole to prevent bacterial infections. Model outcomes included 1 year survival, life expectancy, costs, and incremental cost-effectiveness ratios (ICERs, US$ per quality-adjusted life-year [QALY]); we considered a strategy cost-effective if the ICER was less than $600 per QALY (based on 2023 Malawi per capita gross domestic product).

**Findings:**

ART only resulted in life expectancy of 17·45 undiscounted QALYs and discounted lifetime costs of $1450. All other strategies would increase both QALYs and costs. The WHO-recommended AHD package would result in the greatest life expectancy (19·30 undiscounted QALYs) and be cost-effective (ICER $580 per QALY). AHD prevalence and intervention efficacy had the greatest influence on ICERs; however, the WHO-recommended AHD package would remain cost-effective over a wide range of estimates.

**Interpretation:**

The WHO-recommended AHD package of care at ART initiation would provide substantial clinical benefits and be cost-effective in Malawi. This package for AHD should be made widely available in Malawi and similar settings.

**Funding:**

WHO, the HIV Modelling Consortium within the Institute for Global Health at University College London, the Bill & Melinda Gates Foundation, the National Institute of Allergy and Infectious Diseases, the Massachusetts General Hospital Jerome and Celia Reich Endowed Scholar in HIV/AIDS Research Award, and the Steve and Deborah Gorlin Massachusetts General Hospital Research Scholars Award.

**Translation:**

For the Chichewa translation of the abstract see Supplementary Materials section.

## Introduction

Despite substantial advances in HIV testing and access to antiretroviral therapy (ART), approximately 630 000 people died from AIDS-related causes worldwide in 2023, and UNAIDS mortality reduction targets were not reached.^1^ Many of these deaths occurred in people with advanced HIV disease (AHD), defined by WHO for adults (ie, those older than 19 years) as a CD4 count of less than 200 cells per μL or WHO clinical stage 3 or 4 disease.^2^ People living with HIV who have AHD are at increased risk of opportunistic infections such as tuberculosis, cryptococcal meningitis, and severe bacterial infections, all of which are key drivers of increased mortality.^3^ In sub-Saharan Africa, AHD prevalence ranges from 20% to 40% at the time of ART initiation.^4^, ^5^ Many people living with HIV present to outpatient clinics, at which screening for AHD and testing, treatment, and prevention of associated opportunistic infections could substantially reduce morbidity and improve survival.^5^, ^6^, ^7^

WHO recommends a comprehensive package of care for AHD, including: a CD4 count to screen for AHD in everyone initiating ART, followed by a comprehensive package for people with AHD; rapid ART initiation with adherence support; sputum Xpert MTB/RIF and lateral flow urine lipoarabinomannan (LAM) assay to diagnose tuberculosis; tuberculosis preventive therapy to prevent tuberculosis disease; serum cryptococcal antigen (CrAg) screening and pre-emptive fluconazole therapy if positive to prevent cryptococcal meningitis; and co-trimoxazole prophylaxis to prevent serious bacterial infections.^2^ Despite these recommendations, implementation of the AHD package of care has remained limited due to constrained access to CD4 testing, complexity of the AHD algorithm and decision making, and costs.^8^ Here, we aimed to examine the long-term clinical impact, costs, cost-effectiveness, and budget impact of the WHO-recommended AHD package for people with AHD in Malawi, as an example of one country with substantial resource limitations focused on addressing this challenge.^9^

**Research in context**

**Evidence before this study**

Advanced HIV disease (AHD), defined as a CD4 count of less than 200 cells per μL or WHO clinical stage 3 or 4 disease, is a life-threatening syndrome among people living with HIV who develop severe immunocompromise when not taking antiretroviral therapy (ART). 20–40% of people living with HIV present to clinical care with AHD in sub-Saharan Africa and face an increased risk of death compared with people who present to care without AHD. Two randomised controlled trials established that enhanced prophylaxis and treatment of opportunistic infections could reduce mortality in people living with AHD when initiating ART. In 2017, WHO recommended a comprehensive package of care for people living with AHD, including a CD4 count to diagnose AHD, rapid ART initiation with adherence support, and tuberculosis diagnostics (eg, sputum Xpert and urine lipoarabinomannan), tuberculosis preventive therapy, serum cryptococcal antigen screening with pre-emptive fluconazole treatment if cryptococcal antigen-positive, and co-trimoxazole to prevent bacterial infections. To identify the existing literature related to the cost-effectiveness of screening and treating AHD with the WHO-recommended AHD package of care, we searched PubMed on June 17, 2025, using the search terms: “advanced HIV disease” AND “cost-effectiveness” without language or date restrictions. We identified 10 analyses that assessed a component of the WHO-recommended AHD package of care (eg, cryptococcal screening; tuberculosis diagnostics), and one analysis that compared an enhanced-prophylaxis package versus other strategies for people with AHD, using trial data from the REALITY trial. However, the cost-effectiveness and budget impact of the WHO-recommended AHD package of care has not been examined in settings with substantial resource limitations.

**Added value of this study**

This cost-effectiveness analysis uses the validated Cost-Effectiveness of Preventing AIDS Complications-International simulation model to project the lifetime clinical outcomes, cost-effectiveness, and budget impact of the WHO-recommended AHD package for people living with HIV initiating or reinitiating ART in Malawi. We found that the comprehensive WHO-recommended AHD package, including a CD4 count to diagnose AHD, would result in the greatest life expectancy and would be cost-effective (incremental cost-effectiveness ratio US$580 per quality-adjusted life-year) compared with no availability of the AHD package or only some of the components. The WHO-recommended AHD package would remain cost-effective in Malawi at the cost-effectiveness threshold of $600 per quality-adjusted life-year, even if only 19% of people living with HIV presenting to initiate or reinitiate HIV care had AHD. Among the 80 000 people living with HIV initiating ART in Malawi, the incremental costs over 5 years for the WHO-recommended AHD package compared with ART alone would be $6·98 million, or approximately 2·0% of the 2022–23 Malawi budget for HIV programmes.

**Implications of all the available evidence**

Improving access to comprehensive care for people living with AHD is critical. Previous data from randomised controlled trials and observational cohorts have shown that improving clinical care for people living with AHD reduces mortality, yet incorporation of novel elements such as urine lipoarabinomannan and cryptococcal antigen testing is often not standard in settings with substantial resource limitations. Findings from this model-based analysis show that the WHO-recommended AHD package of care would be cost-effective in Malawi and similar settings in sub-Saharan Africa. Funding to include the WHO-recommended AHD package in routine clinical care for people initiating or reinitiating ART is an excellent investment in health, even in settings with substantial resource limitations.

## Methods

### Analytic overview

We used the Cost-Effectiveness of Preventing AIDS Complications-International (CEPAC-I) microsimulation model^10^ to project clinical outcomes and costs of the WHO-recommended AHD package of care for people in Malawi including: tuberculosis testing, treatment, and prevention; cryptococcal testing and treatment; and co-trimoxazole. We simulated a cohort of people presenting for outpatient ART initiation or reinitiation in Malawi; in ART only*,* all people living with HIV initiate ART at model start without specific screening or treatment for AHD. In the 12 additional strategies, different elements of the AHD package of care were included singly or in combination: sputum Xpert and/or LAM with treatment for tuberculosis, tuberculosis preventive therapy, serum CrAg screening with fluconazole pre-emptive therapy, and co-trimoxazole (appendix 2 p 33). The strategy with all elements of the AHD package of care (ART + Xpert + lipoarabinomannan + co-trimoxazole + CrAg + tuberculosis preventive therapy) is the WHO-recommended AHD package.^2^ We parameterised the model with cohort, clinical trial, and cost data from Malawi and similar sub-Saharan African settings from the health-care payer perspective.^11^ We projected 1 year survival, undiscounted quality-adjusted life-years (QALYs), costs, and cost-effectiveness, as determined by the incremental cost-effectiveness ratio (ICER, or difference in costs/difference in QALYs between strategies, with both costs and QALYs discounted at 3%). We considered strategies with an ICER of less than US$600/QALY (ie, Malawi's 2023 per capita gross domestic product) to be cost-effective and performed sensitivity analyses at different cost-effectiveness thresholds.^12^, ^13^, ^14^ We performed a 5-year budget impact analysis from the payer's perspective.

The research was reviewed and approved by the Mass General Brigham Human Research Committee under Protocol 2014P002708. Any unpublished data are from the Evaluation of Advanced HIV Disease Differentiated Care Model in Malawi study (Principal Investigator TM), which was reviewed and approved by the Malawi National Health Sciences Research Committee (21/06/2720).

### Model structure

The CEPAC-I model is a validated microsimulation of HIV disease progression, treatment, and prevention.^15^, ^16^ People, in the model, living with HIV move between health states based on transition probabilities, stratified by age, sex at birth, current and past CD4 count, HIV RNA and viral set point, history of opportunistic infections, and status of engagement with HIV care. The CEPAC-I model distinguishes between true and observed health states; clinical decisions within the model are based on the person's observed health state, which depends on either symptoms or the results of testing. The model tracks each person from model initiation until death and tallies all clinical events and costs. Additional model details are available in appendix 2 (pp 4–9) and online.^10^

In the model, people living with HIV are subject to CD4-stratified incidence and mortality risks of opportunistic infections, including tuberculosis, cryptococcal disease, and serious bacterial infections, which can co-occur. Each diagnostic test is characterised by acceptance, sensitivity, specificity, and cost. After diagnostic evaluation, people, in the model, living with HIV can initiate treatment or prophylaxis, each with a specified effectiveness and cost.

### Model input parameters

The simulated population includes adults (ie, those aged >19 years) living with HIV presenting or re-presenting to outpatient HIV care in Malawi: 63% are female at birth with a mean age of 33·2 years (SD 9·3) and a mean CD4 count of 330 cells per μL (SD 110; table 1).^4^, ^17^ 25% of the cohort has AHD, including 12·4% with a CD4 count of less than 200 cells per μL and with WHO stage 3 or 4 disease; 8·6% with a CD4 count of less than 200 cells per μL but without WHO stage 3 or 4 disease; and 4·0% with CD4 count of 200 or more cells per μL and WHO stage 3 or 4 disease.^4^, ^18^ People with severe symptoms that would prompt hospitalisation, pregnant people, and children were not included in the simulation, as they represent distinct, important populations.

**Table 1:** Key model parameters to assess the cost-effectiveness of different strategies for the prevention, diagnosis, and treatment of AHD among people living with HIV in Malawi

			**Base case value**
Sex at birth^4^, ^17^			
	Female		63%
	Male		37%
Age, years^4^, ^17^			33·2 (9·3)
Initial CD4 count, cells per μL^4^*			330 (110)
	CD4 count <200 cells per μL with or without WHO stage 3 or 4 disease		90 (50)
	CD4 count ≥200 cells per μL with WHO stage 3 or 4 disease		250 (50)
	No AHD (CD4 count ≥200 cells per μL and no WHO stage 3 or 4 disease)		400 (130)
Cohort distribution			
	CD4 count <200 cells per μL with WHO stage 3 or 4 disease^4^, ^18^		12·4%
	CD4 count <200 cells per μL without WHO stage 3 or 4 disease^4^, ^18^		8·6%
	WHO stage 3 or 4 disease with CD4 count ≥200 cells per μL		4·0%
	No AHD (CD4 count ≥200 cells per μL and no WHO stage 3 or 4 disease)		75·0%
**HIV care continuum**†			
	Virological suppression at 6 months from antiretroviral therapy initiation		
		Integrase strand transfer inhibitor-based regimen^19^, ^20^	92%
		Protease inhibitor-based regimen^21^	73%
	Loss to follow-up over 12 months^22^		8·5%
	Return to care after 12 months of being lost to follow-up, monthly^23^		1·3%
	Return to care upon developing symptoms of new opportunistic infection‡		50%
**Tuberculosis**†			
	CD4-stratified tuberculosis prevalence§		
		Active tuberculosis disease^24^	10–37
		Latent tuberculosis disease^24^	20–47
	Tuberculosis symptoms^25^, ^26^§¶		51–87%
	Monthly active tuberculosis-related mortality among those untreated^7^		7%
	CD4-stratified test diagnostic yield among outpatients with symptoms^27^§‖		
		Xpert	68–70%
		Xpert plus LAM	72–85%
	Test specificity^27^, ^28^		
		Xpert	98%
		Xpert plus LAM	95%
	Probability of receiving empiric tuberculosis treatment^24^§		7–30%
	RHZE efficacy for drug-susceptible-tuberculosis treatment^29^		98%
	Tuberculosis preventive therapy efficacy for preventing tuberculosis disease^30^, ^31^**		43%
**Cryptococcal infection**†			
	Cryptococcal disease prevalence^32^		
		CD4 count <100 cells per μL	7%
		CD4 count 100–200 cells per μL	2%
	Monthly mortality from untreated cryptococcal meningitis^33^		78%
		Cryptococcal antigen test sensitivity^34^	98%
		Cryptococcal antigen test specificity^34^	98%
	Fluconazole efficacy for preventing cryptococcal meningitis^35^, ^36^		72%
**Other opportunistic infections**†			
	Opportunistic infection incidence (stratified by CD4 count and antiretroviral therapy status), monthly§		
		Severe malaria^37^	0·02%
		Serious bacterial infections^7^, ^38^, ^39^, ^40^	0·04–3·68%
		Other WHO stage 3 or 4 disease^38^	0·25–4·59%
	Opportunistic infection mortality		
		Severe malaria^41^	28·1%
		Serious bacterial infections^7^	30·0%
		Other WHO stage 3 or 4 disease^7^	18·7%
	Co-trimoxazole efficacy in preventing incident opportunistic infections		
		Severe malaria^42^, ^43^	88·4%
		Serious bacterial infections^42^, ^43^	49·8%
		Other WHO stage 3 or 4 disease^44^, ^45^	15·0%
**Quality of life, utility**†			
	Age-stratified and sex-stratified^46^§		0·860–0·910
	Tuberculosis^47^		0·620
	Acute opportunistic infection, 1 month		
		Severe malaria^48^	0·52
		Serious bacterial infections^48^	0·54
		Other WHO stage 3 or 4 disease^48^	0·50
	Cryptococcal meningitis, 1 month^48^		0·48
	Major drug toxicity, 1 month^48^		0·75
**AHD care continuum**			
	Percentage of eligible people who have the test performed		
		Sputum Xpert††	79%
		Urine LAM^49^	91%
		Tuberculosis preventive therapy	NA
		Cryptococcal antigen‡	75%
		Co-trimoxazole	NA
	Percentage of people with positive test results who initiate treatment		
		Sputum Xpert^50^	91%
		Urine LAM^50^	91%
		Tuberculosis preventive therapy††	79%
		Cryptococcal antigen‡	90%
		Co-trimoxazole‡	90%
**Costs (2023 USD)**†			
	HIV care		
		TDF–3TC + DTG, monthly^51^	$4
		AZT–3TC + LPV–r, monthly^51^	$19
	AHD care		
		CD4 count, per test^52^	$6
		Sputum Xpert, per test^53^	$16
		Urine LAM, per test^54^	$6
		RHZE treatment, monthly^54^	$12
		Tuberculosis preventive therapy, monthly^54^	$1
		Cryptococcal antigen screening^54^	$4
		Fluconazole pre-emptive therapy, monthly^54^	$5
		Co-trimoxazole prophylaxis, monthly^54^	$1

Data are % or mean (SD) unless otherwise stated. AHD=advanced HIV disease. AZT–3TC + LPV–r=zidovudine and lamivudine with lopinavir–ritonavir. LAM=lateral flow lipoarabinomannan. NA=not applicable. RHZE=rifampicin, isoniazid, pyrazinamide, ethambutol. TDF–3TC + DTG=tenofovir disoproxil fumarate and lamivudine with dolutegravir.

* Values of initial CD4 count are square root transformed (appendix p 4).

† See appendix for additional details (pp 6–9).

‡ Assumption (ie, when no data are available to inform a parameter estimate, then an assumption is made and the estimate in sensitivity analysis is varied.)

§ Range shows input parameters that are stratified by CD4 count, age, and/or sex (appendix pp 18, 20, 23, 25 for additional details).

¶ Based on the WHO-recommended four-symptom screen, comprising current cough, fever, night sweats, and weight loss.

‖ Diagnostic yield, defined as the total proportion of tuberculosis cases identified by the tests; calculated by multiplying the sensitivity of the tests by the proportion of people living with HIV who could provide the diagnostic sample; data from Broger and colleagues.^27^

** Tuberculosis preventive therapy prevents initial tuberculosis infection and the progression of latent tuberculosis infection to active tuberculosis disease with an efficacy of 43% over a period of 30 months; this effect lasts for 24 months after completing tuberculosis preventive therapy.

†† Maphosa T, unpublished data from the Evaluation of Advanced HIV Disease Differentiated Care Model in Malawi study.

We populated the model with derived monthly probabilities of: CD4-stratified, HIV-related mortality, and Malawi-specific age-stratified and sex-stratified, non-HIV-related mortality (table 1; appendix 2 p 18).^38^, ^55^, ^56^ In the model, people living with HIV receive Malawi national guideline-concordant HIV care.^57^ Mean ART adherence is 93%, and loss to follow-up results in 8·5% disengagement from care in the first year.^22^, ^58^

We derived CD4-stratified tuberculosis prevalence, symptom prevalence, quality of life, and mortality, as well as sensitivity and specificity of the tuberculosis diagnostic tests (appendix 2 pp 20–21).^7^, ^24^, ^27^, ^28^, ^47^ We estimated the efficacy of rifampicin, isoniazid, pyrazinamide, and ethambutol for the treatment of drug-susceptible tuberculosis and isoniazid for the prevention of tuberculosis.^29^, ^30^ We derived the CD4-stratified prevalence of asymptomatic cryptococcal antigenaemia, as well as sensitivity and specificity for the serum CrAg screening test (appendix 2 p 22).^32^, ^34^ We estimated mortality from cryptococcal meningitis and the efficacy of pre-emptive fluconazole for preventing progression of asymptomatic cryptococcal disease to cryptococcal meningitis.^33^, ^35^, ^36^ We estimated the CD4-stratified incidence and mortality associated with malaria, severe bacterial infections, and other WHO stage 3 or 4 diseases, and the efficacy of co-trimoxazole to prevent them (appendix 2 p 23).^7^, ^37^, ^38^, ^39^, ^40^, ^42^, ^44^, ^45^

In the base case, 79% of people with tuberculosis symptoms receive sputum Xpert, 91% receive LAM, and 91% initiate rifampicin, isoniazid, pyrazinamide, and ethambutol after a positive test.^49^, ^50^ When tuberculosis preventive therapy is available, 79% of people living with HIV start tuberculosis preventive therapy if tuberculosis treatment is not started (Maphosa T, unpublished data). In strategies with serum CrAg screening and pre-emptive fluconazole, 75% of people living with HIV with a CD4 cell count of less than 200 cells per μL receive a CrAg test; 90% of people with a positive CrAg test initiate pre-emptive fluconazole. In strategies that include co-trimoxazole, 90% of people living with HIV start co-trimoxazole at model start and continue it throughout their lifetime.

We applied quality of life modifiers for CD4-stratified HIV status, tuberculosis, severe malaria, severe bacterial infections, other WHO stage 3 or 4 disease, cryptococcal meningitis, and major drug toxicity (appendix 2 p 25).^47^, ^48^ Costs for CD4 count, Xpert, LAM, and CrAg are $6, $16, $6, and $4 per test, respectively.^52^, ^53^, ^54^ Monthly costs of rifampicin, isoniazid, pyrazinamide, and ethambutol, tuberculosis preventive therapy, fluconazole pre-emptive treatment, and co-trimoxazole are $12, <$1, $5, and <$1, respectively.^54^ Additional costs are shown in appendix 2 (p 24).

### Sensitivity analysis

To examine how parameter uncertainty affects the results, we performed univariate and bivariate sensitivity analyses on key parameters, including the prevalence of AHD, the percent of eligible people who are recommended for testing and then initiate treatment, and the benefit of the adherence support.^59^ For each univariate sensitivity analysis, we calculated the net monetary benefit, defined as the product of the QALY gain and the cost-per-QALY cost-effectiveness threshold, minus the incremental cost to determine the preferred strategy at different cost-effectiveness thresholds ranging from $260 to $650/QALY, which includes previously determined cost-effectiveness thresholds for ART use ($320 per disability-adjusted life-year [DALY]), tuberculosis preventive therapy use ($650/DALY), and Xpert use ($650/DALY) in Malawi.^13^ In bivariate sensitivity analyses, we varied the parameters with the greatest impact in univariate sensitivity analysis and determined the preferred strategy at the cost-effectiveness threshold of $600/QALY.

### Scenario analyses

Because CD4 testing is not available in all settings, the WHO-recommended AHD package includes guidance for screening and treatment when CD4 counts are unknown.^2^ We compared all strategies recommended when CD4 testing is and is not available to examine the value of CD4 testing at ART initiation. In settings without a CD4 count performed at ART initiation, clinical criteria (ie, WHO clinical stage 3 or 4 disease) guide the use of the AHD package of care and as many as 34% of people with AHD will not be identified as having AHD.^4^ We did not include CrAg screening in strategies without a CD4 test because CrAg is not recommended in the absence of CD4 testing.^2^ Because a CD4 count of less than 200 cells per μL is often used to prioritise people living with HIV for tuberculosis testing and other AHD-focused elements of care, as well as rapid ART initiation, we performed two scenario analyses in which CD4 count is not available: reduced implementation of the AHD package of care (ie, Xpert [50% *vs* 79%], LAM [38% *vs* 91%], tuberculosis preventive therapy [39% *vs* 79%], and co-trimoxazole [60% *vs* 90%]; appendix 2 p 42)^60^, ^61^ and delayed ART initiation for people without WHO stage 3 or 4 disease, which could occur in the setting of stockouts where people with undiagnosed AHD might not be prioritised for ART initiation. In both scenarios, we compared seven strategies without CD4 testing and 13 strategies with CD4 testing (appendix 2 p 9).

### Budget impact analysis

To examine the budget impact of the AHD package of care, we projected undiscounted costs over 5 years among 80 000 people living with HIV estimated to initiate ART in Malawi in 1 year, of whom 25% had AHD.^4^, ^18^, ^62^ We stratified costs by ART, HIV routine care, and the different elements of the AHD package of care from the payer perspective.^62^

### Role of the funding source

The funders of the study had no role in study design, data collection, data analysis, data interpretation, or writing of the report.

## Results

**Figure 1:**
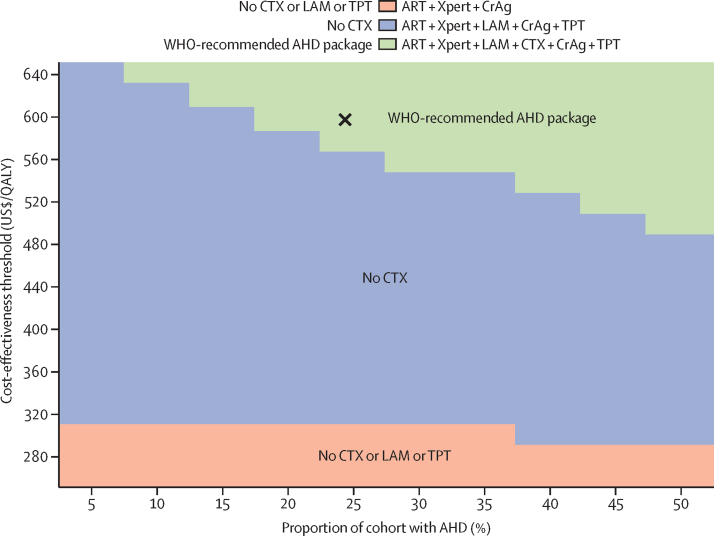
Scenario assessing how the proportion of the cohort initiating or reinitiating ART with AHD impacts the cost-effectiveness of different strategies for the prevention, diagnosis, and treatment of AHD among people living with HIV in Malawi The preferred strategy depends on the proportion of the cohort with AHD (horizontal axis) and the cost-effectiveness threshold (vertical axis) and is shown in the colour defined in the legend, where the strategy components are shown to the right of the colour box and details explaining the missing components of the WHO-recommended AHD package in the strategy are to the left of the box. The base case (ie, the most likely scenario) is marked with a black X. AHD=advanced HIV disease. ART=antiretroviral therapy. CrAg=cryptococcal antigen. CTX=co-trimoxazole. LAM=urine lipoarabinomannan. QALY=quality-adjusted life year. TPT=tuberculosis preventive therapy.

In all people initiating or reinitiating ART, ART only would result in 1 year survival of 91·56%, life expectancy of 17·45 undiscounted QALYs, and total lifetime costs of $1450 (table 2). All AHD strategies would increase both life expectancy and costs compared with ART only. The WHO-recommended AHD package (Xpert + LAM + co-trimoxazole + CrAg + tuberculosis preventive therapy) would provide the greatest benefit, increasing survival at 1 year by 1·98 percentage points and undiscounted QALYs by 1·85, and would be cost-effective (ICER $580/QALY) at a threshold of $600/QALY. Compared with ART only, the WHO-recommended AHD package would reduce 1 year tuberculosis deaths by 30%, cryptococcal meningitis deaths by 25%, and deaths from severe bacterial infections by 38%. When examining the clinical impact of the WHO-recommended AHD package only on people with AHD, clinical benefits would be greater, increasing 1 year survival by 5·06 percentage points and life expectancy by 2·33 undiscounted QALYs. Each component of the AHD package would improve lifetime survival with relatively modest additional cost; three strategies had ICERs that represented an efficient use of resources compared with ART only: $170/QALY (Xpert + CrAg), $310/QALY (Xpert +  LAM + CrAg + tuberculosis preventive therapy), and $580/QALY (WHO-recommended AHD package; appendix p 38). The WHO-recommended AHD package is the preferred choice because it would offer the most clinical benefit with an ICER below the cost-effectiveness threshold. In populations for which 19% or more of the people initiating or reinitiating ART had AHD, the WHO-recommended AHD package would remain preferred at a cost-effectiveness threshold of $600/QALY (figure 1). At higher cost-effectiveness thresholds than $600/QALY, the WHO-recommended AHD package remains the preferred strategy as long as more than 10% of the proportion of the cohort has AHD, whereas at lower cost-effectiveness thresholds (eg, $540/QALY), at least 40% of the cohort must have AHD for the WHO-recommended AHD package to be the preferred strategy.

**Table 2:** Model-projected clinical outcomes, costs, and cost-effectiveness of different strategies for the prevention, diagnosis, and treatment of advanced HIV disease among people living with HIV in Malawi

		1 year decrease in deaths*(%)			**1 year survival**†**(%)**	**Undiscounted QALYs**	**Discounted QALYs**‡	**Discounted lifetime costs (US$)**†‡	**ICER($/QALY)**§¶
		Tuberculosis (%)	Cryptococcal meningitis (%)	Serious bacterial infections (%)					
All individuals presenting for outpatient ART initiation or reinitiation									
	ART only	..	..	..	91·56%	17·45	11·28	1450	..
	+ Xpert	25%	(2%)	(1%)	92·74%	18·48	11·81	1540	Dominated
	**+ Xpert + CrAg**	**25%**	**28%**	**(1%)**	**92·75%**	**18·48**	**11·81**	**1540**	**170**
	+ Xpert + LAM	28%	(3%)	(2%)	92·88%	18·67	11·90	1580	Dominated
	+ Xpert + LAM + CrAg	28%	27%	(1%)	92·89%	18·67	11·90	1580	Dominated
	+ Xpert + LAM + TPT	31%	(2%)	(1%)	93·02%	18·76	11·96	1590	Dominated
	**+ Xpert + LAM + CrAg + TPT**	**31%**	**25%**	**(1%)**	**93·05%**	**18·76**	**11·96**	**1590**	**310**
	+ Xpert + CTX	24%	(3%)	38%	93·23%	19·01	12·09	1700	Dominated
	+ Xpert + CTX + CrAg	24%	26%	38%	93·24%	19·02	12·09	1700	Dominated
	+ Xpert + LAM + CTX	27%	(4%)	38%	93·36%	19·21	12·18	1740	Dominated
	+ Xpert + LAM + CTX + CrAg	27%	26%	38%	93·38%	19·22	12·18	1740	Dominated
	+ Xpert + LAM + CTX + TPT	30%	(4%)	38%	93·52%	19·31	12·24	1750	Dominated
WHO-recommended advanced HIV disease package		**30%**	**25%**	**38%**	**93·54%**	**19·30**	**12·24**	**1750**	**580**

ART=antiretroviral therapy. CrAg=cryptococcal antigen. CTX=co-trimoxazole. ICER=incremental cost-effectiveness ratio. LAM=lateral flow lipoarabinomannan. QALY=quality-adjusted life year. TPT=tuberculosis preventive therapy.

* Each strategy compared with ART only clinical outcomes. All values are a decrease in deaths at 1 year, unless in parentheses, which signifies an increase in deaths at 1 year; additional deaths can occur due to competing risks of mortality (eg, when fewer people die due to tuberculosis, there can be an increase in deaths due to serious bacterial infections). Bolded strategies offer the greatest value compared with the others.

† The percentage of the cohort that survives 1 year in the model.

‡ Discounted at 3% per year.

§ Reported total costs and ICERs are rounded to the nearest $10.

¶ Strategies are dominated if an intervention has a higher ICER than that of another intervention that provides more QALYs. We report undiscounted health outcomes but use discounted clinical and cost outcomes to calculate ICERs, as recommended by the Second Panel on Cost-effectiveness in Health and Medicine.^63^

In other univariate sensitivity analyses, the WHO-recommended AHD package would remain the preferred strategy across a wide range of parameter estimates at a cost-effectiveness threshold of $600/QALY (appendix 2 pp 27–28). Results are most sensitive to estimates of the prevention and treatment of cryptococcal and severe bacterial infections (appendix 2 pp 40–41). The WHO-recommended AHD package would remain preferred if cryptococcal prevalence is 5% or greater, linkage to pre-emptive fluconazole after positive CrAg test is 80% or greater, efficacy of pre-emptive fluconazole is 70% or greater, and if CrAg costs less than $6 and monthly fluconazole pre-emptive therapy costs less than $9. The WHO-recommended AHD package would remain preferred if the efficacy of co-trimoxazole to prevent severe bacterial infections is greater than 40%, the incidence of severe bacterial infections is the same or greater as previously reported, if average mortality from severe bacterial infections is greater than 22%, or if the monthly cost of co-trimoxazole is less than or the same as current estimates.^54^ The WHO-recommended AHD package would remain cost-effective for tuberculosis parameters at a cost-effectiveness threshold of $600/QALY. Preferred strategies at cost-effectiveness thresholds less than $600/QALY are shown in appendix 2 (pp 39–41).

**Figure 2:**
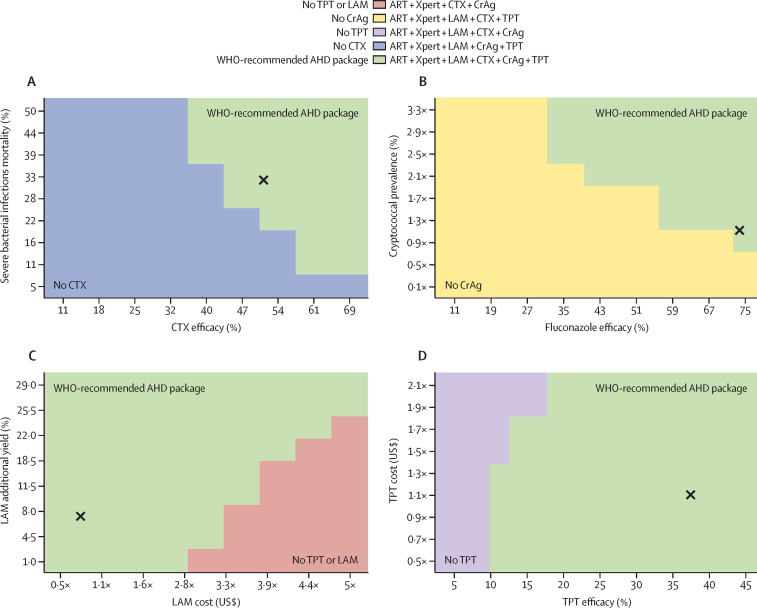
Selected bivariate sensitivity analyses to assess the cost-effectiveness of different strategies for the prevention, diagnosis, and treatment of AHD among people living with HIV in Malawi at a cost-effectiveness threshold of US$600/QALY Each panel represents a bivariate sensitivity analysis. The horizontal and vertical axes show the range of each parameter varied and the most cost-effective strategy is shown in colour for each combination of parameters. The results are shown for a cost-effectiveness threshold of $600/QALY. The strategy components are shown to the right of the coloured key; details to the left explain the missing components of the WHO-recommended AHD package in the strategy. The base case is marked with a black X. The WHO-recommended AHD package (green) is the most frequently preferred strategy, despite the wide variation in input parameters. AHD=advanced HIV disease. ART=antiretroviral therapy. CrAg=cryptococcal antigen. CTX=co-trimoxazole. LAM=urine lipoarabinomannan. QALY=quality-adjusted life-year. TPT=tuberculosis preventive therapy.

In bivariate sensitivity analyses, we examined the impact of changes in four pairs of parameters that are influential in univariate sensitivity analyses (figure 2). The WHO-recommended AHD package would remain preferred at a cost-effectiveness threshold of $600/QALY, unless co-trimoxazole efficacy is 47% or lower and mortality due to severe bacterial infections is 28% or lower (figure 2A). If pre-emptive fluconazole efficacy is greater than 67% and if cryptococcal prevalence is at least 90% of current estimates^32^, ^35^ (figure 2B), the WHO-recommended AHD package would be preferred. Additionally, the WHO-recommended AHD package would be preferred unless there is only a 1% improvement in diagnostic yield with LAM testing added to Xpert and LAM cost is $20 per test or more (figure 2C). The WHO-recommended AHD package also would not be preferred when tuberculosis preventive therapy efficacy is less than 10%, regardless of the prevalence of latent tuberculosis disease (figure 2D).

The WHO-recommended AHD package, which includes CD4 count at ART initiation, would remain preferred at a cost-effectiveness threshold of $600/QALY, even when compared with all strategies without a CD4 count available at ART initiation (appendix 2 pp 29–30, 42). When implementation of the WHO-recommended AHD package is lower among people living with HIV without WHO stage 3 or 4 disease, strategies that include CD4 counts would be preferred unless the cost-effectiveness threshold is less than $200/QALY (appendix 2 pp 29, 42). When ART initiation is delayed by 1 month in people living with HIV without WHO stage 3 or 4 disease, strategies that include CD4 counts would be preferred unless the cost-effectiveness threshold is less than $130/QALY (appendix 2 pp 30, 42).

**Figure 3:**
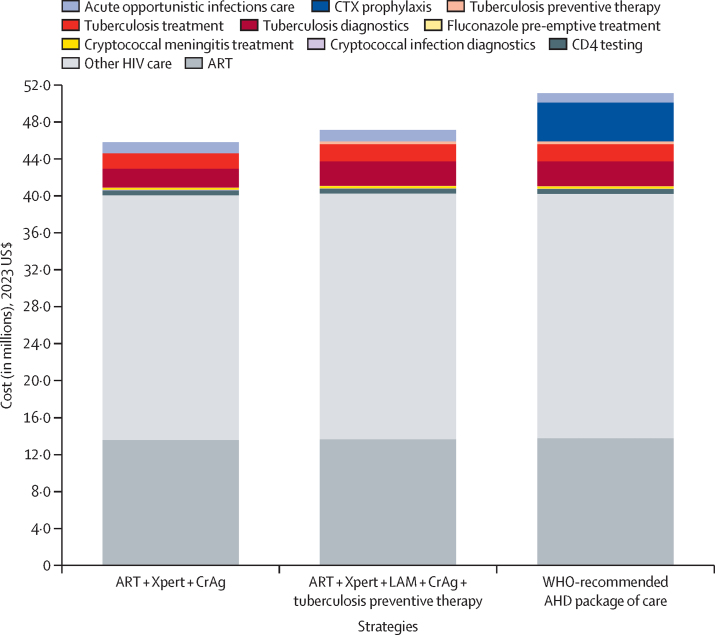
Budget impact analysis for the WHO-recommended AHD package of care for people living with HIV and initiating or reinitiating ART in Malawi Each bar represents all HIV-related costs accrued over the first 5 years of HIV clinical care for 80 000 outpatients living with HIV initiating or reinitiating ART in Malawi, of whom 25% have AHD. The majority of costs are due to ART and HIV clinical care (gray). AHD=advanced HIV disease. ART=antiretroviral therapy. CrAg=cryptococcal antigen. CTX=co-trimoxazole. LAM=urine lipoarabinomannan.

In a simulated cohort of 80 000 people living with HIV initiating outpatient ART in Malawi, the total costs over 5 years would range from $44·21 million with ART only to $51·19 million with the WHO-recommended AHD package from the payer perspective (figure 3). Incremental costs over 5 years for the WHO-recommended AHD package compared with ART only included: $1·76 million for tuberculosis testing; $182 000 for tuberculosis treatment; and $302 000 for tuberculosis preventive therapy; $44 000 for cryptococcal infection screening; $37 000 for fluconazole pre-emptive treatment; and $4·20 million for co-trimoxazole (figure 3; appendix 2 p 43). The WHO-recommended AHD package would reduce care costs for severe bacterial infections and other co-trimoxazole-preventable opportunistic infections by $167 000 and costs for cryptococcal meningitis treatment by $74 000. Total incremental costs over 5 years for the WHO-recommended AHD package compared with ART only would be $6·98 million, with $6·28 million directed towards the AHD package of care and $0·84 million for increased ART and HIV care costs due to improved survival. This additional investment is equivalent to 2·0% of Malawi's 2022–23 budget for HIV programmes provided by external funders (ie, the US President's Emergency Plan for AIDS Relief and The Global Fund to Fight AIDS, Tuberculosis and Malaria) or 2·5% of the Government of Malawi's total health sector funding for the fiscal year 2022–23.^62^, ^64^

## Discussion

In this model-based analysis, we found that the WHO-recommended AHD package of care, including sputum Xpert and LAM for tuberculosis diagnosis, tuberculosis preventive treatment, serum CrAg screening with pre-emptive fluconazole therapy, and co-trimoxazole, in addition to rapid ART initiation, would improve quality-adjusted life expectancy, reduce the incidence of many highly morbid opportunistic infections, and be cost-effective in Malawi. Although the AHD package of care is cost-effective, it would not be cost-saving, since extending life for people living with HIV means that they will incur the costs of clinical care and ART for the extended time in their lives. This combined package would offer substantial survival benefits, particularly during the first year after ART initiation, when people living with HIV have the highest mortality risk, and would reduce deaths due to tuberculosis, cryptococcal meningitis, and serious bacterial infections. If at least 19% of people living with HIV presenting to initiate or reinitiate care have AHD, then implementing the WHO-recommended AHD package of care would provide high value at a cost-effectiveness threshold of $600/QALY.

The WHO-recommended AHD package of care would remain the preferred strategy in extensive sensitivity analyses at a cost-effectiveness threshold of $600/QALY, the 2023 Malawi per capita gross domestic product, compared with ART initiation alone or different elements of the AHD package singly or in combination. Even at lower cost-effectiveness thresholds, the full AHD package of care would remain preferred if the incidence of and mortality from opportunistic infections, efficacy of prevention and treatment interventions, and costs were within plausible estimates as have been reported. Only at extremely low cost-effectiveness thresholds would the preferred strategy include some combination of elements in the package but not the full package of care. This analysis showed that incorporation of at least some elements of the WHO-recommended AHD package of care would be cost-effective at any cost-effectiveness threshold greater than $170/QALY. Even with a lack of consensus about what might be an appropriate cost-effectiveness threshold in low-income and middle-income countries, $580/QALY could be a valuable use of limited resources.

We found that the WHO-recommended AHD package of care would be cost-effective and would require a budget increase or redistribution of approximately 1·8% in funding allocated to the HIV response in Malawi in 2022–23. In a budget impact analysis, we projected that incremental costs of incorporating the WHO-recommended AHD package of care for a cohort of 80 000 people initiating ART in Malawi would total $6·98 million over 5 years, of which $6·28 million would be directed to the AHD package of care and $0·69 million to ART and routine HIV clinical care for people living with HIV who would live longer because of the AHD package of care. Given the 2022–23 HIV Malawi budget by external funders (ie, the US President's Emergency Plan for AIDS Relief and The Global Fund) of $352 million per year and the Government of Malawi health sector spending budget of $274 million for 2022–23, investment in the WHO-recommended AHD package of care would require an increase or redistribution of 1·1% in all health-care spending.^62^, ^64^

There has been a decreased prioritisation of CD4 count tests in the HIV Treat All era, based on the idea that CD4 counts might not be needed because the results no longer determine ART eligibility, and HIV RNA testing is now prioritised for treatment monitoring.^65^ Our analysis highlights the important role of CD4 count testing in prioritising resources for people living with HIV who might have advanced disease but are without clinical symptoms or signs at the time of presentation for ART initiation or reinitiation. This is an important use of CD4 count testing that is distinct from treatment monitoring. These model-based results show that a reduction in implementation of the AHD package of care, or even a 1-month delay in ART initiation for people not clinically recognised as having AHD, would result in substantially worse clinical outcomes. These findings are similar to those in a modelling analysis showing the clinical benefits of including CD4 testing at clinical presentation to guide the implementation of tuberculosis and cryptococcal disease testing and treatment.^66^ Including a CD4 count at ART initiation identifies the substantial number of people at high risk for morbidity and mortality and for whom clinical services should be prioritised; these services include both ART and the WHO-recommended AHD package of care.^67^ These findings underscore the need to ensure access to CD4 testing.

These model-based results are directly applicable to Malawi and are likely to be generalisable to other settings faced with substantial resource limitations. Results are consistent across a wide range of plausible input parameters for tuberculosis, cryptococcal disease, and serious bacterial infection incidences, which can vary across settings. Thus, this analysis highlights that investments in the WHO-recommended AHD package of care would offer good value even in settings with an uncertain prevalence of tuberculosis, cryptococcal meningitis, and serious bacterial infections. Implementation studies are also needed to understand the barriers to access and to determine best practices for how to incorporate the different elements of the WHO-recommended AHD package into ART initiation in a variety of settings. Additionally, the diagnostics, treatments, and prophylaxes that comprise the WHO-recommended AHD package are not always procured by recipients of international funding, although such expenditures can be supported by The Global Fund.^62^, ^68^ These results show that funding to include these elements in clinical care will be a cost-effective investment even in settings with substantial resource limitations that have not incorporated these additional testing modalities previously.

This study has several limitations. Although we used the best available data to populate the model for clinical, quality-of-life, and cost parameters, there is always uncertainty and variability in data estimates, as well as the potential for model misspecification bias. We performed extensive sensitivity analyses to assess the effect of data uncertainty on the policy conclusions and found that the WHO-recommended AHD package of care would remain cost-effective in Malawi under most plausible ranges of estimates. Although we incorporated most of the important costs of the AHD package, such as shipping, taxes, and labour, when estimating the per-test costs of each element of the package, there could be additional capital investments and training that were not included.^69^ Some countries have different policies regarding the AHD package of care (eg, reflex CrAg testing in a centralised laboratory) that we did not examine. Additionally, CD4 testing can misspecify people with AHD and could be useful for serial measurement among people who experience medication toxicity;^70^ the implications of different CD4 tests or testing strategies could be assessed in future analyses. We focused on outpatients and did not assess the impact of the WHO-recommended AHD package of care for people who are hospitalised and living with HIV initiating ART.

The WHO-recommended AHD package of care at ART initiation, including a CD4 count to identify people most immunocompromised, would improve health outcomes and be cost-effective in Malawi, with a modest increase in total costs of care. Efforts to scale up the WHO-recommended AHD package of care and expand opportunities for tuberculosis diagnosis, treatment, and prevention; screening for asymptomatic cryptococcal disease and prevention of cryptococcal meningitis; and prevention of serious bacterial infections with co-trimoxazole should be more widely implemented.

### Contributors

### Data sharing

No new data were collected for this study. All data used as model inputs are publicly available and can be accessed from the sources cited in the Article and appendix with the exception of unpublished data from the Elizabeth Glaser Pediatric AIDS Foundation, which are available upon request to Thulani Maphosa (tmaphosa@pedaids.org). These include AHD care continuum data, specifically the percentage of eligible people who will have Sputum Xpert performed and the percentage of people with positive test results who will initiate tuberculosis preventive treatment.

## Declaration of interests

AP has received support from the National Institute for Health Research, the US National Institutes of Health, the Wellcome Trust, and the EU. CRH is a member of the Board of Directors of the International Union Against Tuberculosis and Lung Disease. All other authors declare no competing interests.

## Supplementary Materials

Chichewa translation of the abstract
