# Supplementary material for: Clinical impact and cost-effectiveness of the WHO-recommended advanced HIV disease package of care
Source: Lancet Glob Health. 2025 Jul 22;13(8):e1436–47. doi: 10.1016/S2214-109X(25)00190-1 (PMC12286912; doi:10.1016/S2214-109X(25)00190-1)
Supplement: Chichewa translation of the abstract [file mmc1.pdf]

THE LANCET
Global Health

Supplementary appendix 1

This translation in Chichewa was submitted by the authors and we reproduce it as supplied. It has not been peer reviewed. *The Lancet*'s editorial processes have only been applied to the original in English, which should serve as reference for this manuscript.

Kutanthauzila kwa mu Chichewa uku kwachokela kwa alembi a nkhaniyi ndipo tayipeleka kwa inu m'mene tinayilandilila. Mawu a mu Chichewa sanawunikilidwenso kapena kukonzedwa. Nkhani ya mu Chingelezi yokha ndi imene yadutsa mu ukonzi wa Lancet, kotelo kuti nkhani ya mu Chingeleziyi ndi imene ikuyimilila mokwanila nkhani yonse imene yalembedwa.

Supplement to: Hyle EP, Maphosa T, Rangaraj A, et al. Clinical impact and cost-effectiveness of the WHO-recommended advanced HIV disease package of care. *Lancet Glob Health* 2025; **13:** e1436–47.

Hyle EP, Maphosa T, Rangaraj A, Feser M, Singini GC, Shrestha P, Shroufi A, Reddy KP, Matiya E, Dambe R, Talbot VR, Chamanga R, Horsburgh RC, Weinstein MC, Nyirenda RK, Ford N, Tiam A, Phillips A, Freedberg KA. Clinical impact and cost-effectiveness of the WHO-recommended advanced HIV disease (AHD) package of care. *Lancet Global Health.* 2025. In press.

CHIDULE CHA KAFUKUFUKU

<u>Chiyambi cha kafukufuku</u>: Kumwela kwa chipululu cha sahara,pakati pa anthu 20 mpaka 40 pa 100 aliwonse amene ali ndi HIV amakhala odwalika kwambiri chifukwa cha HIV (AHD), zomwe zitha kufufuzidwa ndikupezeka,kuthandizidwa ndikutetezedwa pogwilitsa ntchito phukusi lachisamaliro chomwe chinavomerezedwa ndi a bungwe lowona zaumoyo padziko lonse lapansi (WHO). Tinawunikila mtengo wabwino komaso momwe phukusi lachisamaliro cha anthu odwalika ndi HIV (AHD) lomwe ndi lovomerezeka ndi owona za umoyo padziko lonse lapasi lingakhudzile chuma choyendetsela dziko kuno ku Malawi.

<u>Ndodomeko Ya Kafukufuku</u>

Kugwilitsa ntchito njila ya CEPAC-International model, tinayelekeza gulu la anthu amene anapezeka ndi kachilombo ka HIV (>18y) osagonekedwa kuchipatala amene amayambitsidwa mankhwala a HIV ;; anthu 25 pa 100 aliwonse anali wodwalika ndi HIV (chitetezo chotsika kwambiri kapena amadwala matenda akulu kutengela ndi oyang'anila za matenda pa dziko lapansi (WHO) Tinawunikila njila 13 zokhazikika,kupatikiza mankhwala a ART okha mpakana

Phukusi lovomelezeka ndi a WHO la anthu odwalika ndi HIV,kuphatikiza kayezedwe ka chifuwa chachikulu(monga , kuyeza makhololo,Mkodzo,mankhwala oteteza kutengela chifuwa chachikulu,kuyeza chizindikilo za matenda oumitsa khosi kudzala mmagazi ndi kulandila mankhwala a fulukonazo ngati atapezeka ndi kuti ali ndi matenda owumitsa khosiwa, ndi kumwa mankhwala a Bakitirimu oteteza matenda oyambitsidwa ndi tizilombo ta bakiteria mthupi. Zotsatila zikuphatikiza chaka chimodzi chokhala ndi moyo,choyembekezeledwa ndi moyo,Mitengo,ndipo njira ya zoyeza zakasamalidwe ka chuma (ICER , ICER, $/quality-adjusted life-year [QALY]); tinawona njirayi kuti ndiyosamala chuma ngati ICER <$600/kuti ukhale ndi moyo wathanzi (2023 Malawi *per capita* GDP)

Zotsatira : *Mankwhala ama ARV okha angapangise munthu kukhala ndi moyo kwa pafupifupi zaka khumi ndi zisanu ndi ziwiri (17.4), moyo wathanzi wotsatsika komanso kuchotsera mtengo wamoyo wonse wandalama zokwana 1 450 za dziko la America.* . Njira zina zonse zitha kuonjezera Chaka Chamoyo Chokhazikika (QALY) ndi zolipila .. . Phukusi lothandizila anthu omwe akudwala kwambili chifukwa cha HIV/Aids (AHD) lomwe linavomerezedwa ndi bungwe lowona zaumoyo padziko lonse (WHO) lingapangitse kuti moyo ukhale wautali kwambiri (zaka 19.3) komanso wotsika mtengo(ICER,$580/QALY) . .. Kuchuluka kwa anthu odwalika chifukwa cha HIV (AHD)ndi kagwilidwe ntchito kazithandizo kunakhudza kwambiri Mitengo; komabe, Phukusi la AHD lovomerezedwa ndi bungwe lowona zaumoyo padziko lonse lapansi (WHO) lakhalabe lotsikilapo mtengo pakuwunikila zambiri.

<u>Mapeto a kafukufuku</u>: Phukusi la chithandizo cha anthu odwalika ndi HIV lovomerezeka ndi bungwe la zaumoyo padziko lonse lapasi (WHO) lopelekedwa panthawi yoyamba kumwa mankhwala a ART itha kukhala yopindulitsa kwambiri kuumoyo komaso kukhala. Phukusi ili la anthu wodwalika ndi HIV likuyenela kupezeka madela ambiri Mmalawi ndi maiko ena ofananila.
