# Supplementary material for: Clinical impact and cost-effectiveness of the WHO-recommended advanced HIV disease package of care
Source: Lancet Glob Health. 2025 Jul 22;13(8):e1436–47. doi: 10.1016/S2214-109X(25)00190-1 (PMC12286912; doi:10.1016/S2214-109X(25)00190-1)
Supplement: Supplementary appendix 2 [file mmc2.pdf]

THE LANCET
Global Health

Supplementary appendix 2

This appendix formed part of the original submission and has been peer reviewed. We post it as supplied by the authors.

Supplement to: Hyle EP, Maphosa T, Rangaraj A, et al. Clinical impact and cost-effectiveness of the WHO-recommended advanced HIV disease package of care. *Lancet Glob Health* 2025; **13:** e1436–47.

Clinical impact and cost-effectiveness of the

WHO-recommended advanced HIV disease (AHD) package of care

Supplementary Appendix

Emily P. Hyle
Thulani Maphosa
Ajay Rangaraj
Mary Feser
Amir Shroufi
Geoffrey C. Singini
Prakriti Shrestha
Krishna P. Reddy
Eddie Matiya
Rosalia Dambe
Virginia R. Talbot
Rachel Chamanga
C. Robert Horsburgh
Milton C. Weinstein
Rose K. Nyirenda
Nathan Ford
Appolinaire Tiam
Andrew Phillips
Kenneth A. Freedberg

CONTENTS

SUPPLEMENTARY METHODS 4

CEPAC-I module structure 4

TB module 4

Cryptococcus module 5

Serious bacterial infections, other WHO clinical stage 3 or 4 diseases, and severe malaria 5

Model input parameterization 6

Input parameters 6

Model validation 9

Scenario analyses 9

SUPPLEMENTARY RESULTS 10

Model validation 10

Univariate sensitivity analyses 10

REFERENCES 11

SUPPLEMENTARY TABLES AND FIGURES 18

Table S1. Detailed input parameters for HIV natural history. 18

Table S2. Detailed input parameters for HIV treatment and care continuum. 19

Table S3. Detailed input parameters for natural history, diagnosis, treatment, and prevention of TB. 20

Table S4. Detailed input parameters for the natural history, diagnosis, treatment, and prevention of cryptococcal infection. 22

Table S5. Detailed input parameters for the natural history, diagnosis, treatment, and prevention of severe malaria, serious bacterial infections, and other WHO stage 3/4 diseases. 23

Table S6. Detailed costs parameters (USD 2023). 24

Table S8. Cohort characteristics among PLWH initiating standard care in REALITY trial validation(13). 26

Table S9. Model-projected cost-effectiveness results for the WHO-recommended AHD package of care in univariate sensitivity analysis. 27

Table S10. Model-projected clinical outcomes, costs, and cost-effectiveness of different strategies for the prevention, diagnosis, and treatment of AHD among PLWH in Malawi who experience lower uptake of AHD package in absence of a CD4 count. 29

Table S11. Model-projected clinical outcomes, costs, and cost-effectiveness of different strategies for the prevention, diagnosis, and treatment of AHD among PLWH in Malawi who experience a one-month delay in ART initiation in absence of a CD4 count. 30

SUPPLEMENTARY FIGURE LEGENDS 31

Figure S1. Schematic of the elements in the *WHO-recommended AHD Package*. 33

Figure S2. Overview of TB module health states and transitions. 34

Figure S3. Overview of the cryptococcus module health states and transitions. 35

Figure S4. CEPAC-I projections of mortality compared with observed mortality in the REALITY trial. 36

Figure S5. CEPAC-I projected cause of death compared with REALITY trial results for people initiating ART with AHD. 37

Figure S6. Model-projected incremental lifetime clinical outcomes and costs for all strategies with CD4 test available. 41

Figure S7. Univariate sensitivity analyses for selected clinical and cost parameters over a range of cost-effectiveness thresholds... 41

Figure S8. Model-projected incremental lifetime clinical outcomes and costs for all strategies with and without a CD4 test available in two scenarios 42

Figure S9. Detailed budget impact analysis for the *WHO-recommended AHD Package* for people living with HIV in Malawi. 43

SUPPLEMENTARY METHODS

CEPAC-I model structure

CEPAC-I is a computer-based, state-transition, Monte Carlo microsimulation model of HIV disease progression, treatment, and prevention (1). The model simulates a cohort of patients by drawing from user-specified distributions of age, sex at birth, CD4 cell count, HIV RNA viral set point, and adherence to HIV care. To avoid the potential problem of drawing a negative value for CD4, the input parameters for initial CD4 count are square-root transformed; the model draws from a normal distribution of the square root of the CD4 cell count, with the values for that distribution determined so that they match a mean and standard deviation from the published literature and listed in Table 1. During the simulation, individuals experience monthly probabilities of experiencing different health events that are based on published data and stratified by clinically relevant characteristics (e.g., current CD4 cell count, viral suppression status). These parameters are varied in sensitivity analysis.

The model traces clinical outcomes and costs as each simulated person transitions through these different states of disease progression and treatment. Model details, including flowcharts and descriptions of health states and transition probabilities, are available online (https://mpec.massgeneral.org/cepac-model/).

Simulated PLWH receive Malawi national guideline-concordant HIV care, including a CD4 count and first-line ART (tenofovir disoproxil fumarate and lamivudine with dolutegravir [TLD]), treatment monitoring with HIV RNA measurement annually, and switch to a protease inhibitor (PI)-based regimen if diagnosed with virologic failure (2).

TB module

TB natural history

The TB module of the CEPAC-I model includes the following health states: Uninfected, Latent TB, Active TB, Previously Treated TB, and TB Treatment LTFU (Figure S2) (3). These five TB states reflect the true state of the simulated person, which could be different than the clinically observed state. Transition probabilities between health states depend on user-defined probabilities of TB infection, reactivation, treatment completion (i.e., treatment success or failure), relapse, and loss to follow-up (LTFU). People in the Active TB state are subject to a monthly probability of dying from TB if untreated.

Simulated people living with HIV (PLWH) have a possibility of TB symptoms (i.e., current cough, fever, weight loss, or night sweats) at model start based on user-defined probabilities and a probability of developing TB symptoms each subsequent month; both differ based on the true health state (e.g., people with active TB have a higher probability of TB symptoms than people who do not have active TB).

TB diagnostics

PLWH with TB symptoms are eligible for TB diagnostic test(s): the sputum-based GeneXpert MTB/RIF assay (Xpert) with or without the lateral flow urine lipoarabinomannan assay (LAM). Each test has its own user-specified sensitivity and specificity. The user also specifies the probability of providing a sputum specimen for Xpert testing, given that not all people are able to do so (4). PLWH with TB symptoms can also receive standard-of-care (SOC) test(s), which include a chest X-ray (CXR) with or without sputum smear microscopy. The user defines a probability that PLWH eligible for a TB diagnostic test will actually undergo that test.

TB treatment

TB treatment can be initiated in two situations: 1) empirically for people with TB symptoms who never have Xpert/LAM testing or who receive a negative Xpert/LAM result; 2) for people who receive a positive Xpert/LAM result.

TB treatment (i.e., 6-month regimen of Rifampin, Isoniazid, Pyrazinamide, and Ethambutol [RHZE]) is associated with a user-defined probability of treatment efficacy (cure upon completing the 6-month regimen, after which the individual transitions to the Previously Treated TB state) and with a monthly cost. People who do not attain cure remain in the Active TB state and are subject to mortality risks from TB. TB treatment is associated with a monthly probability of toxicity, which includes a small probability of death from toxicity and costs associated with treating toxicity. People who inappropriately initiate TB treatment when they are not in the Active TB state (e.g., due to

inaccurate use of empiric treatment or a false positive TB diagnostic test result) remain in their true health state while incurring the toxicity risks and costs of TB treatment.

PLWH are at risk of being LTFU during TB treatment. If this occurs, the individual then has a probability of treatment success (cure) proportional to the number of months of treatment they have completed. PLWH who attain cure while LTFU will transition to the TB Treatment LTFU state wherein they are not subject to mortality from untreated TB. PLWH who do not attain cure remain in the Active TB state.

TB prevention

PLWH are eligible for TB preventive therapy (TPT) either if they have no TB symptoms, or if they do not receive a positive diagnostic TB test and are not selected for empiric treatment. TPT reduces the probability of TB infection, reinfection, or reactivation (i.e., progression from latent infection to active disease) for a user-specified period. People with undiagnosed active TB who are receiving TPT with isoniazid (INH) have a 50% probability of developing INH resistance (5). Those who develop INH resistance will face a lower probability of TB treatment success, if they subsequently initiate RHZE treatment for TB disease. TPT is associated with a monthly probability of toxicity that leads to its discontinuation and the incurrence of toxicity-related costs.

<u>Cryptococcus module</u>

Natural history

We developed a novel cryptococcus module that simulates the natural history, diagnosis, treatment, and prevention of *C. neoformans* infection (Figure S3). When PLWH become infected with *C. neoformans*, they enter the Cryptococcemia state (i.e., asymptomatic infection in the bloodstream only). Individuals with asymptomatic cryptococcemia have a user-defined probability of progression from cryptococcemia to cryptococcal meningitis, wherein they transition to the Symptomatic Meningitis state; this probability can vary based on time since infection and receipt of antifungal medications. PLWH with cryptococcal meningitis have cryptococcal infection in the meninges and brain tissue and experience central nervous system (CNS) symptoms and increased risk of mortality.

Cryptococcal diagnostics

PLWH in any cryptococcal infection health state can be diagnosed with either cryptococcemia or cryptococcal meningitis and initiated on antifungal medications. Diagnosis of cryptococcemia occurs when an asymptomatic person receives a positive serum cryptococcal antigen (CrAg) test (Se 97.6%, Sp 98.1%) (6). Patients with symptoms consistent with cryptococcal meningitis can receive lumbar puncture (LP) and cerebrospinal fluid (CSF) CrAg testing. Diagnosis of cryptococcal meningitis occurs upon a positive CSF CrAg test (2).

Cryptococcal treatment

Per Malawi national guidelines, PLWH with a positive CrAg test and no meningitis symptoms initiate fluconazole preemptive therapy to prevent progression to cryptococcal meningitis (2). PLWH diagnosed with cryptococcal meningitis initiate intensive antifungal treatment; any treatment toxicity that results in mortality is included in the mortality associated with treated cryptococcal meningitis. When an LP is infeasible or refused, people with symptoms consistent with cryptococcal meningitis can initiate intensive antifungal treatment based on clinical suspicion (empiric treatment). After successful preemptive fluconazole therapy or cryptococcal meningitis treatment, PLWH will transition to the Previously Treated Cryptococcemia or Previously Treated Symptomatic Meningitis health states respectively. PLWH can experience relapse/reinfection while in these states. PLWH can become LTFU at any stage of cryptococcus testing, diagnosis, or treatment.

<u>Serious bacterial infections, other WHO clinical stage 3 or 4 diseases, and severe malaria</u>

Serious bacterial infections (SBIs), other WHO clinical stage 3 or 4 diseases, and severe malaria are acute opportunistic infections (OIs); within the CEPAC-I model, their incidence, treatment, and mortality occur in a one-month timeframe defined by CD4-stratified monthly probabilities. ART further reduces the CD4-stratified OI incidence. In the month of the incident OI, PLWH experience an increased risk of mortality and costs associated with treatment. The occurrence of an OI does not reduce the probability of that OI in future.

PLWH who are taking OI prophylaxis, such as co-trimoxazole (CTX), have a reduced probability of SBIs, other WHO stage 3/4 diseases, and severe malaria. There are costs associated with OI prophylaxis and toxicities that occur while taking OI prophylaxis.

Model input parameterization

Input parameters

HIV

Table S1 includes the detailed model input parameters for HIV natural history. Table S2 includes the detailed input parameters for HIV treatment and care continuum.

TB natural history

Among people with CD4 <200 cells/µL, active TB prevalence ranges from 18%-37%, while latent TB prevalence is 20%-39% and 43% of people do not have TB infection (Table S3) (7–9). Active TB is considered a WHO stage 3/4 disease (10). We simulated PLWH with symptomatic active TB and non-symptomatic active TB (subclinical TB) in the sub-cohorts with and without WHO stage 3/4 disease, respectively. We assumed that the total proportion of PLWH with any TB infection (latent or active) is unaffected by the presence or absence of a WHO 3/4 disease and remains constant across all CD4 strata. After dividing PLWH with active TB into the two sub-cohorts based on their symptom status, we back calculated the proportion of PLWH with latent TB for both sub-cohorts across all CD4 strata. In the sub-cohort with WHO stage 3/4 disease, the CD4-stratified estimated prevalence was 14%-51% for active TB and 6%-43% for latent TB. Similarly, in the sub-cohort without WHO stage 3/4 disease, active TB prevalence was 6%-13% and latent TB prevalence was 44%-51%.

Eighty-seven percent of people with active TB and CD4 <200 cells/µL present with at least one TB symptom whereas 67% without active TB and CD4 <200 cells/µL present with at least one symptom consistent with active TB (11). Of PLWH infected with TB, 99% have the drug susceptible strain (DS-TB) and the remaining 1% have a drug-resistant strain (8). We also derived CD4-stratified incidence of TB infection and TB reactivation (Table S3) (12,13). Mortality from untreated, active TB is 7% per month (14).

TB diagnostic inputs

PLWH who develop symptoms of TB in subsequent months can receive TB testing at clinic visits. Seventy-nine percent of PLWH eligible for an Xpert test accept it, and 87% could produce sputum (15,16). Similarly, 91% of people offered a LAM test accept it (16). We assumed that 100% of PLWH would be able to produce urine.

Diagnostic yield (DY) was defined as the number of TB cases correctly identified by a testing strategy (accounting for the ability to obtain a sample) divided by the number of true TB cases according to a study-defined reference standard (4). Among ambulatory PLWH with TB symptoms, GeneXpert MTB/RIF has a DY of 68%-70% (4). Adding LAM to strategies with Xpert provided an additional yield of 2%-17% (i.e., DY, 72%-85%, when both Xpert and LAM are used (4).

TB empiric treatment

PLWH with TB symptoms can also be diagnosed via chest X-ray with or without sputum smear or may initiate TB treatment empirically (RHZE regimen) without any diagnostic testing (8). This may occur if: Xpert or LAM are not available, not performed on the patient, or a negative Xpert or LAM result is received. In settings where Xpert/LAM are available, 7-17% of symptomatic PLWH who do not have Xpert/LAM performed or who test negative will initiate treatment empirically; in settings without available Xpert/LAM, 12-30% of PLWH with TB symptoms initiate TB treatment empirically (8).

TB treatment

Of people diagnosed with active TB disease, 91% link to TB treatment, and 88% complete treatment if they remain in care (17). Among PLWH who complete TB treatment, 98% will be cured if they have drug-susceptible TB and 69% will be cured if they have drug-resistant TB (18). Toxicities of TB medications can occur with associated impact on quality of life and costs (19). We assume that PLWH who are LTFU from HIV care will also be LTFU from TB care.

TB preventive therapy (TPT) efficacy calibration

To capture the impact of TPT in CEPAC-I, we calibrated the efficacy of a 6-month TPT regimen using data from TEMPRANO, a randomized controlled trial of early ART and TPT (isoniazid) that reported the reduction in TB diagnoses among people who received both ART and TPT compared with people who received only ART (13). We

simulated the TEMPRANO study using characteristics of the study population: mean age 36y, mean CD4 472 cells/µL (SD 140 cells/µL), latent TB prevalence 33% (based on an interferon-gamma release assay [IGRA]). No one with suspected active TB qualified for the trial and anyone who developed TB symptoms received a diagnostic test. Loss to follow-up at the end of trial was 3.50% and 2.51% in the no TPT and TPT groups, respectively.

We assumed that when including all reported TB diagnoses (definite, probable, and possible) from the study, the diagnostic algorithm in TEMPRANO captured nearly all true TB cases. We calibrated the probabilities of TB progression to match the reported proportion of TB cases from TEMPRANO.

TPT prevents initial TB infection and the progression of latent TB infection to active TB disease with an efficacy of 43% over a period of 30 months; this effect lasts for 24 months after completing TPT (14). Among eligible PLWH, 79% initiate TPT when offered, and 87% complete it (Maphosa T, EGPAF, unpublished, 22).

Cryptococcal disease
CD4-stratified prevalence and incidence of cryptococcemia are outlined in Table S4, as well as progression from asymptomatic cryptococcemia to symptomatic meningitis, which occurs only in PLWH with CD4 <200 cells/µL (22,23). Without fluconazole preemptive therapy, progression from asymptomatic cryptococcal antigenemia to symptomatic cryptococcal meningitis is 100% without ART, which decreases to 30% over 6 months, if ART is initiated (24,25). Given the need to simplify the complex pathophysiology of cryptococcal disease for the simulation model, we do not explicitly model quantitative CrAg titers. One-month mortality from symptomatic meningitis is 78% without antifungal treatment (26).

Serum CrAg has a sensitivity of 97.6% and specificity of 98.1% in asymptomatic PLWH with 95% uptake of testing (6,27). Of PLWH with a positive CrAg test, 90% start on preemptive fluconazole. For PLWH diagnosed with cryptococcemia, fluconazole preemptive therapy consists of 800 mg/day fluconazole for two weeks followed by 400 mg/day for 8 weeks (2). PLWH are then maintained on 200 mg/day of fluconazole for 9 months; we simulated a total treatment course of one year. Efficacy of preemptive fluconazole is 72% against progression from asymptomatic cryptococcemia to symptomatic meningitis (25).

For PLWH with symptomatic cryptococcal meningitis, we assume that 90% will have access to an LP. Among them, 73% will undergo LP with CSF CrAg for diagnostic testing (28). Of people with cryptococcal meningitis diagnosed by LP, 98% will start cryptococcal meningitis treatment. If an LP is not obtained, 88% of people with meningitis symptoms will empirically start cryptococcal meningitis treatment. Meningitis treatment occurs in three phases: induction, consolidation, and maintenance. Recommended induction therapy is a single dose of liposomal amphotericin B (10 mg/kg via IV), with flucytosine (100 mg/kg/day for 14 days) and fluconazole (1200 mg/day for 14 days). The consolidation phase includes 800 mg/day fluconazole for 8 weeks followed by the maintenance phase which includes 200 mg/day of fluconazole 9 months (2). Treatment does not differ between PLWH who were diagnosed via LP and CSF testing versus people initiating empiric treatment. The preferred alternative regimen, if liposomal amphotericin B is not available, is flucytosine and fluconazole administered orally for 14 days (2). PLWH will be lost to follow-up from cryptococcal infection care if they are lost to follow-up from HIV care.

Serious bacterial infections and other OIs
Table S5 shows monthly incidence for SBIs (0.03%-3.68%) and for other WHO stage 3/4 diseases (0.20%-4.59%), stratified by CD4 count and ART status (14,29–32). Severe malaria incidence is 0.024% (33–36) among PLWH not treated with ART and 0.017% (37) for PLWH on ART; it is not CD4-stratified. Mortality in the month of infection is 30.0% for SBIs, 18.7% other WHO stage 3/4 diseases, and 28.1% for severe malaria (14,33).

Calibration of serious bacterial infection (SBI) incidence
Prior CEPAC analyses used incidence and mortality estimates for SBIs derived from the Cape Town AIDS Cohort (CTAC) study (1992-2000) (29). Because availability of ART and improved diagnostics for SBIs could affect diagnosed OI incidence and OI-related mortality, we validated CTAC inputs at higher CD4 counts to the Strategic Timing of Antiretroviral Therapy (START) trial and calibrated CTAC-derived incidence inputs at lower CD4 counts to the REALITY trial, which are two randomized controlled trials that provide recent, detailed data on SBIs in PLWH in sub-Saharan Africa (14,30,38).

We validated SBI incidence among PLWH with CD4 >500 cells/µL against data from the START trial (38). The START trial was a randomized controlled trial in ART-naïve PLWH with CD4 counts >500 cells/µL that evaluated the impact of immediate versus deferred ART start. We calculated the cumulative incidence of non-tuberculosis SBIs among African PLWH in the immediate ART start arm; 0.62% of the starting population experienced an OI over the study period (median follow-up time, 2.8 years; IQR, 2.1-3.9) (30). We then parameterized CEPAC with a cohort that resembled the African START cohort in age, sex at birth, CD4 count, and symptom status and compared SBI incidence from CEPAC to SBI incidence from START. In the simulated population with mean CD4 count >500 /µL, the monthly probability of SBI incidence (when on-ART) derived from CTAC (0.03%/month) projected a cumulative incidence of SBIs similar to the START trial (0.7% of the initial population experienced an SBI over 2 years).

We calibrated incidence at lower CD4 counts using published incidence rates from the Reduction of Early Mortality in HIV-Infected Adults and Children Starting Antiretroviral Therapy (REALITY trial), a factorial open-label trial that took place from 2013-2015 in Uganda, Zimbabwe, Malawi, and Kenya (14). The trial evaluated the impact of enhanced prophylaxis compared to co-trimoxazole alone. Using rates published in a secondary trial analysis (31), we derived a monthly probability of SBI incidence among people with CD4 counts 50-100 cells/µL on ART: 1.50%. We then used incidence estimates at CD4 counts 50-100 cells/µL and >500 cells/µL and the relationship between SBI incidence and CD4 count from the CTAC data to extrapolate SBI at other CD4 counts. Using the calibrated incidence inputs, CEPAC projections of SBI incidence closely matched REALITY trial results. We calculated mortality within one month of an SBI to be 30.0%, using deaths caused by new, presumptive cases of SBI from REALITY (14).

Other WHO clinical stage 3 or 4 diseases calculations
We derived the incidence of other WHO clinical stage 3 or 4 diseases from CTAC data, which includes all other WHO stage 3/4 diseases, excluding TB, cryptococcal meningitis, and SBIs. We calculated mortality of other WHO stage 3/4 diseases using data from the REALITY trial (14).

Malaria incidence and mortality calculations
We calculated a monthly probability of severe malaria incidence using data on age-stratified malaria deaths and a published infection fatality rate for severe malaria in adults (33,34). We assumed that all malaria deaths occurred in individuals with severe malaria prior to death. We then adjusted the probability using a rate multiplier of 2.41 to account for the increased likelihood of severe malaria among PLWH compared to the general population (39).

Quality of life
We incorporated quality of life (QoL) decrements for opportunistic infections, including TB, CM, SBI, malaria, among other opportunistic infections (Table S7) (40). We also incorporated a QoL decrement for major toxicity from TB treatment of half the weight of the general QoL input, as patients hospitalized for major toxicity from TB treatment are hospitalized for approximately half the mean duration of admission for PLWH on ART (7 days versus 12.2 days, respectively) (40). QoL estimates for living with HIV were CD4-stratified and incorporated in the model (41).

Costs
We obtained costs for diagnostic tests and drugs from Songane and colleagues, including test costs for LAM, chest X-ray, and CrAg and drug costs for isoniazid, co-trimoxazole, fluconazole, flucytosine, and liposomal amphotericin B (42). Lab monitoring and labor costs (i.e., nurse and doctor time) were obtained from EGPAF Malawi. We acquired the cost of a sputum smear test from Maheswaran and colleagues (40). We calculated costs for antiretroviral and TB treatment drugs, as well as Xpert, CD4 count, and HIV viral load testing using *ex works* prices from technical briefs, catalogs, and pricing reports from the Global Fund, Clinton Health Access Initiative (CHAI), and others (43–48). We included an 25% additional cost on top of the *ex works* prices, for international shipping and handling (49). For labor costs associated with performing any diagnostic tests, we accounted for 15 minutes of nurse time at $3.29/hour (15). We used estimates of the cost of lumbar puncture with CSF CrAg and added 30 minutes each of physician and nurse time (15,50).

We estimated the costs of one hospitalized day (stratified by OI type) and an outpatient visit (40,51). We calculated the outpatient visit cost by summing the average cost of visiting an HIV clinic in Malawi (non-clinical personnel

time, equipment, space, overhead, et cetera) with 20 minutes of nurse time (15,51). For CD4-stratified monthly routine care costs and costs associated with treating OIs and death, we used resource utilization data in terms of the average number of inpatient days and outpatient visits required (29). We multiplied these data with hospitalized day and outpatient visit costs respectively. We used the Malawi inflation index and the average 2023 exchange rate to convert all costs to 2023 U.S. dollars (36,52). Detailed cost input parameters are reported in Table S6.

Model validation

AHD validation and calibration in CEPAC-I

We validated CEPAC model outcomes against published data from the REALITY trial for people with AHD initiating ART. We populated CEPAC-I with cohort characteristics that closely matched participants in the REALITY trial (Table S8): mean age 36y, 53.8% male, and mean CD4 36/µL (SD, 33/µL)(14). At model start, 15% are diagnosed with active TB and 1.3% are diagnosed with cryptococcal meningitis. Annual loss to follow-up is 3.5%.

Scenario analyses

CD4 testing is not available

We examined scenarios in which CD4 count testing is not available in the assessment of AHD in PLWH initiating ART. Of the cohort, 12.4% have both CD4 <200/µL and a WHO stage 3/4 disease, while 8.6% have CD4 <200/µL but no WHO stage 3/4 disease (53,54). The latter group would therefore not be diagnosed with AHD in the absence of a CD4 count. CrAg testing is also not available at model start in scenarios without a CD4 count given the WHO recommendations do not include CrAg testing to people with an unknown CD4 (55). Therefore, we assessed the following strategies from the *WHO-recommended AHD Package* when CD4 count testing is not available: ART Only, ART+Xpert, ART+Xpert+LAM, ART+Xpert+LAM+TPT, ART+Xpert+CTX, ART+Xpert+LAM+CTX, ART+Xpert+LAM+CTX+TPT.

We assessed the impact of not having a CD4 count available with two different scenarios:

A) Lower uptake of AHD package of care elements among PLWH without an AHD diagnosis

In addition to the unavailability of CrAg testing at model start, we assumed that the probability of sputum Xpert testing uptake would decrease from 79% to 50%, while uptake of urine LAM would decrease from 91% to **38**% (56). Similarly, TPT uptake would decrease from 79% to 39% (57). We assumed that co-trimoxazole uptake would decrease from 90% to 60% among PLWH without an AHD diagnosis.

B) One month ART initiation delay among PLWH without an AHD diagnosis

PLWH with an AHD diagnosis initiate ART at model start while PLWH without an AHD diagnosis only initiate ART after a one-month delay.

3HP as TPT

We also performed a scenario analysis in which TPT is provided as 3HP instead of 6H. Input parameters for the 3HP scenario included: efficacy of 3HP (i.e., reduction in probability of progression from tuberculosis infection to tuberculosis disease) (49%) (58,59); regimen duration (3 months); probability of major hepatoxicity (0.011%/month) (59); an average monthly cost of $4.91 (44). We assumed the same uptake of 3HP as IPT (79%) given the absence of data regarding 3HP uptake in people with HIV in Malawi or sub-Saharan Africa outside of clinical trials. We did not examine 1HP as it is not frequently in use (20).

SUPPLEMENTARY RESULTS

Model validation

CEPAC projections of all-cause mortality at 48 weeks were similar to all-cause mortality reported in REALITY (14.2% mortality in CEPAC vs 14.4% mortality in REALITY) (Figure S4) (14). CEPAC-projected causes of death were aligned with causes of death reported in REALITY (Figure S5) (14).

Univariate sensitivity analyses

Among TB-related parameters, LAM testing with Xpert would be cost-effective when LAM increases TB diagnostic yield by 1% or more, at a cost-effectiveness threshold of $260-360/QALY. Similarly, TPT would be cost-effective to include in the *WHO-recommended AHD Package* at a TPT efficacy of >5% (when the cost-effectiveness threshold is $480/QALY) or at >10% (when the cost-effectiveness threshold is $300-400/QALY). The *WHO-recommended AHD Package* would remain the preferred strategy except when the prevalence of cryptococcal infection is <5% (at a cost-effectiveness threshold of $600/QALY). Similarly, CrAg would be cost-effective even when the efficacy of preemptive fluconazole is only 50% (at a cost-effectiveness threshold of $360/QALY), or linkage to preemptive fluconazole is less than 40% (at a cost-effectiveness threshold of $320/QALY). The *WHO-recommended AHD Package* would also remain the preferred strategy over a wide range of costs for the different interventions, even at cost-effectiveness thresholds much lower than $600/QALY. The impact of the *WHO-recommended AHD Package* on adherence was not extremely influential; when adherence was 93% in the AHD cohort but only 85% for people without AHD, the ICER was essentially unchanged.

3HP Scenario Analysis

Compared with *ART alone*, the *WHO-recommended AHD Package (Xpert+LAM+CTX+CrAg+TPT)* would be the preferred strategy, increasing 1y survival by 1.98 percentage points, undiscounted (discounted) QALYs by 1.89 (0.97), and discounted costs by $300. The *WHO-recommended AHD Package* would be cost-effective, with an ICER of $540/QALY (at a cost-effectiveness threshold of $600/QALY).

REFERENCES

1. Luz PM, Girouard MP, Grinsztejn B, Freedberg KA, Veloso VG, Losina E, et al. Survival benefits of antiretroviral therapy in Brazil: A model-based analysis. J Int AIDS Soc. 2016;19(1).

2. Clinical management of HIV in children and adults [Internet]. Lilongwe, Malawi: Ministry of Health and Population; 2022 [cited 2023 Jun 7]. Available from: https://www.differentiatedservicedelivery.org/wp-content/uploads/Malawi-Clinical-HIV-Guidelines-2022-edition-5.pdf

3. Reddy KP, Gupta-Wright A, Fielding KL, Costantini S, Zheng A, Corbett EL, et al. Cost-effectiveness of urine-based tuberculosis screening in hospitalised patients with HIV in Africa: a microsimulation modelling study. Lancet Glob Health. 2019 Feb;7(2):e200–8.

4. Broger T, Koeppel L, Huerga H, Miller P, Gupta-Wright A, Blanc FX, et al. Diagnostic yield of urine lipoarabinomannan and sputum tuberculosis tests in people living with HIV: a systematic review and meta-analysis of individual participant data. Lancet Glob Health. 2023 Jun 1;11(6):e903–16.

5. Egsmose T, Ang'awa JOW, Poti SJ. The use of isoniazid among household contacts of open cases of pulmonary tuberculosis. Bull World Health Organ. 1965;33(3):419–33.

6. Huang HR, Fan LC, Rajbanshi B, Xu JF. Evaluation of a new cryptococcal antigen lateral flow immunoassay in serum, cerebrospinal fluid and urine for the diagnosis of cryptococcosis: a meta-analysis and systematic review. PLoS One. 2015;10(5):e0127117.

7. Dodd PJ, Millington KA, Ghani AC, Mutsvangwa J, Butterworth AE, Lalvani A, et al. Interpreting tuberculin skin tests in a population with a high prevalence of HIV, tuberculosis, and nonspecific tuberculin sensitivity. Am J Epidemiol. 2010 May 1;171(9):1037–45.

8. Henostroza G, Harris JB, Chitambi R, Siyambango M, Turnbull ER, Maggard KR, et al. High prevalence of tuberculosis in newly enrolled HIV patients in Zambia: need for enhanced screening approach. Int J Tuberc Lung Dis. 2016 Aug;20(8):1033–9.

9. Lawn SD, Brooks SV, Kranzer K, Nicol MP, Whitelaw A, Vogt M, et al. Screening for HIV-associated tuberculosis and rifampicin resistance before antiretroviral therapy using the Xpert MTB/RIF assay: a prospective study. PLoS Med. 2011 Jul;8(7):e1001067.

10. Annex 10: WHO clinical staging of HIV disease in adults, adolescents and children [Internet]. Consolidated guidelines on the use of antiretroviral drugs for treating and preventing HIV infection: recommendations for a public health approach. 2nd edition. World Health Organization; 2016 [cited 2025 Mar 18]. Available from: https://www.ncbi.nlm.nih.gov/books/NBK374293/

11. Dhana A, Hamada Y, Kengne AP, Kerkhoff AD, Rangaka MX, Kredo T, et al. Tuberculosis screening among ambulatory people living with HIV: a systematic review and individual participant data meta-analysis. Lancet Infect Dis. 2022 Apr 1;22(4):507–18.

12. Dodd PJ, Looker C, Plumb ID, Bond V, Schaap A, Shanaube K, et al. Age- and sex-specific social contact patterns and incidence of Mycobacterium tuberculosis infection. Am J Epidemiol. 2016 Jan 15;183(2):156–66.

13. TEMPRANO ANRS 12136 Study Group, Danel C, Moh R, Gabillard D, Badje A, Le Carrou J, et al. A trial of early antiretrovirals and isoniazid preventive therapy in Africa. N Engl J Med. 2015 Aug 27;373(9):808–22.

14. Hakim J, Musiime V, Szubert AJ, Mallewa J, Siika A, Agutu C, et al. Enhanced prophylaxis plus antiretroviral therapy for advanced HIV infection in Africa. N Engl J Med. 2017 Jul 20;377(3):233–45.

15. Advanced HIV Disease (AHD) study data, EGPAF Malawi. 2023.

16. Ku CC, MacPherson P, Khundi M, Nzawa Soko RH, Feasey HRA, Nliwasa M, et al. Durations of asymptomatic, symptomatic, and care-seeking phases of tuberculosis disease with a Bayesian analysis of prevalence survey and notification data. BMC Med. 2021 Nov 10;19:298.

17. Lungu P, Kerkhoff AD, Kasapo CC, Mzyece J, Nyimbili S, Chimzizi R, et al. Tuberculosis care cascade in Zambia - identifying the gaps in order to improve outcomes: a population-based analysis. BMJ Open. 2021 Aug 10;11(8):e044867.

18. Espinal MA, Kim SJ, Suarez PG, Kam KM, Khomenko AG, Migliori GB, et al. Standard short-course chemotherapy for drug-resistant tuberculosis: treatment outcomes in 6 countries. JAMA. 2000 May 17;283(19):2537–45.

19. Yee D, Valiquette C, Pelletier M, Parisien I, Rocher I, Menzies D. Incidence of serious side effects from first-line antituberculosis drugs among patients treated for active tuberculosis. Am J Respir Crit Care Med. 2003 Jun 1;167(11):1472–7.

20. Rosen LV, Thielking AM, Dugdale CM, Montepiedra G, Kalk E, Kim S, et al. Tuberculosis Preventive Treatment for Pregnant People With Human Immunodeficiency Virus in South Africa: A Modeling Analysis of Clinical Benefits and Risks. Clin Infect Dis. 2024 Nov 15;ciae508.

21. World Health Organization. Global tuberculosis report 2022: TB prevention [Internet]. [cited 2025 Mar 18]. Available from: https://www.who.int/teams/global-tuberculosis-programme/tb-reports/global-tuberculosis-report-2022/tb-prevention

22. Rajasingham R, Smith RM, Park BJ, Jarvis JN, Govender NP, Chiller TM, et al. Global burden of disease of HIV-associated cryptococcal meningitis: an updated analysis. Lancet Infect Dis. 2017 Aug 1;17(8):873–81.

23. Ford N, Shubber Z, Jarvis JN, Chiller T, Greene G, Migone C, et al. CD4 cell count threshold for cryptococcal antigen screening of HIV-infected individuals: a systematic review and meta-analysis. Clin Infect Dis. 2018 Mar 4;66(Suppl 2):S152–9.

24. Jarvis JN, Lawn SD, Vogt M, Bangani N, Wood R, Harrison TS. Screening for cryptococcal antigenemia in patients accessing an antiretroviral treatment program in South Africa. Clin Infect Dis. 2009 Apr 1;48(7):856–62.

25. Temfack E, Bigna JJ, Luma HN, Spijker R, Meintjes G, Jarvis JN, et al. Impact of routine cryptococcal antigen screening and targeted preemptive fluconazole therapy in antiretroviral-naive human immunodeficiency virus–infected adults with CD4 cell counts <100/μL: a systematic review and meta-analysis. Clin Infect Dis. 2019 Feb 1;68(4):688–98.

26. Heyderman RS, Gangaidzo IT, Hakim JG, Mielke J, Taziwa A, Musvaire P, et al. Cryptococcal meningitis in human immunodeficiency virus-infected patients in Harare, Zimbabwe. Clin Infect Dis. 1998 Feb;26(2):284–9.

27. Enock K, Julius K, Griffith BC, Abila DB, Rutakingirwa MK, Kasibante J, et al. Evaluation of the initial 12 months of a routine cryptococcal antigen screening program in reduction of HIV-associated cryptococcal meningitis in Uganda. BMC Health Serv Res. 2022 Mar 4;22(1):301.

28. Nalintya E, Meya DB, Lofgren S, Huppler Hullsiek K, Boulware DR, Rajasingham R. A prospective evaluation of a multisite cryptococcal screening and treatment program in HIV clinics in Uganda. J Acquir Immune Defic Syndr 1999. 2018 Jun 1;78(2):231–8.

29. Holmes CB, Wood R, Badri M, Zilber S, Wang B, Maartens G, et al. CD4 decline and incidence of opportunistic infections in Cape Town, South Africa: implications for prophylaxis and treatment. J Acquir Immune Defic Syndr. 2006 Aug 1;42(4):464–9.

30. O'Connor J, Vjecha MJ, Phillips AN, Angus B, Cooper D, Grinsztejn B, et al. Effect of immediate initiation of antiretroviral therapy on risk of severe bacterial infections in HIV-positive people with CD4 cell counts of more than 500 cells per µL: secondary outcome results from a randomised controlled trial. Lancet HIV. 2017 Mar;4(3):e105–12.

31. Post FA, Szubert AJ, Prendergast AJ, Johnston V, Lyall H, Fitzgerald F, et al. Causes and timing of mortality and morbidity among late presenters starting antiretroviral therapy in the REALITY trial. Clin Infect Dis. 2018 Mar 4;66:S132–9.

32. Balachandra S, Rogers JH, Ruangtragool L, Radin E, Musuka G, Oboho I, et al. Concurrent advanced HIV disease and viral load suppression in a high-burden setting: Findings from the 2015–6 ZIMPHIA survey. PLoS One. 2020 Jun 25;15(6):e0230205.

33. Dondorp AM, Lee SJ, Faiz MA, Mishra S, Price R, Tjitra E, et al. The relationship between age and the manifestations of and mortality associated with severe malaria. Clin Infect Dis. 2008 Jul 15;47(2):151–7.

34. Roser M, Ritchie H. Malaria [Internet]. Our world in data. 2024 [cited 2025 Mar 18]. Available from: https://ourworldindata.org/malaria

35. World malaria report 2021 [Internet]. Geneva: World Health Organization; 2021. Available from: https://www.who.int/publications-detail-redirect/9789240040496

36. World Bank Open Data [Internet]. [cited 2025 Mar 18]. World Bank Open Data. Available from: https://data.worldbank.org

37. Losina E, Yazdanpanah Y, Deuffic-Burban S, Wang B, Wolf LL, Messou E, et al. The independent effect of highly active antiretroviral therapy on severe opportunistic disease incidence and mortality in HIV-infected adults in Côte d'Ivoire. Antivir Ther. 2007;12(4):543–51.

38. Lundgren JD, Babiker AG, Gordin F, Emery S, Grund B, Sharma S, et al. Initiation of antiretroviral therapy in early asymptomatic HIV infection. N Engl J Med. 2015 Aug 27;373(9):795–807.

39. Mahittikorn A, Kotepui KU, De Jesus Milanez G, Masangkay FR, Kotepui M. A meta-analysis on the prevalence and characteristics of severe malaria in patients with Plasmodium spp. and HIV co-infection. Sci Rep. 2021 Aug 17;11(1):16655.

40. Maheswaran H, Petrou S, Cohen D, MacPherson P, Kumwenda F, Lalloo DG, et al. Economic costs and health-related quality of life outcomes of hospitalised patients with high HIV prevalence: A prospective hospital cohort study in Malawi. PLoS One. 2018 Mar 15;13(3):e0192991.

41. Resch SC, Foote JHA, Wirth KE, Lasry A, Scott JA, Moore J, et al. Health impact and cost-effectiveness of HIV testing, linkage, and early antiretroviral treatment in the Botswana Combination Prevention Project. J Acquir Immune Defic Syndr 1999. 2022 Aug 1;90(4):399–407.

42. Songane M, Mukherjee S, Chamanga R, Maphosa T, Longwe B, Namathanga J, et al. Cost of Providing Advanced HIV Disease Treatment Services through Malawi's Hub-and-Spoke Model. Am J Trop Med Hyg. 2024 Oct 2;111(4):897–903.

43. CHAI antiretroviral (ARV) benchmark price comparison list, version: Q2 2022 [Internet]. Clinton Health Access Initiative; 2022 [cited 2025 Mar 18]. Available from: https://www.newhivdrugs.org/post/chai-arv-benchmark-price-comparison-list

44. Global drug facility (GDF) medicines catalog - October 2023 [Internet]. Stop TB Partnership/Global Drug Facility; 2023 [cited 2024 Apr 9]. Available from: https://www.stoptb.org/sites/default/files/gdf_medicines_catalog_5.pdf

45. Médecins Sans Frontières (MSF). MSF access campaign technical brief; time for $5: GeneXpert diagnostic tests. MSF Access Campaign; 2019.

46. The Global Fund. Pooled procurement mechanism reference pricing: RDTs (version: Q4 2023) [Internet]. The Global Fund; 2023 [cited 2025 Feb 20]. Available from: https://www.theglobalfund.org/media/7564/psm_hivrdtreferencepricing_table_en.pdf

47. Global fund agreements substantially reduce the price of first-line HIV treatment to below US$45 a year [Internet]. The Global Fund; 2023 Aug [cited 2025 Mar 18]. Available from: https://www.theglobalfund.org/en/news/2023/2023-08-30-global-fund-agreements-substantially-reduce-price-first-line-hiv-treatment-below-usd45-a-year

48. African Society for Laboratory Medicine. African Society for Laboratory Medicine. 2023 [cited 2025 Mar 18]. Molecular diagnostic pricing database. Available from: https://aslm.org/diagnostic-pricing-database/

49. Larson B, Shroufi A, Muthoga C, Oladele R, Rajasingham R, Jordan A, et al. An Excel-based template for estimating induction-phase treatment costs for cryptococcal meningitis in high HIV-burden African countries [Internet]. 2021 [cited 2022 May 3]. Available from: https://open.bu.edu/handle/2144/41876

50. Rajasingham R, Rolfes MA, Birkenkamp KE, Meya DB, Boulware DR. Cryptococcal meningitis treatment strategies in resource-limited settings: a cost-effectiveness analysis. PLoS Med. 2012;9(9):e1001316.

51. Maheswaran H, Petrou S, MacPherson P, Kumwenda F, Lalloo DG, Corbett EL, et al. Economic costs and health-related quality of life outcomes of HIV treatment after self- and facility-based HIV testing in a cluster randomized trial. J Acquir Immune Defic Syndr. 2017 Jul 1;75(3):280–9.

52. International Financial Statistics. Exchange Rates selected indicators [Internet]. [cited 2025 Jan 8]. Available from: https://data.imf.org

53. de Waal R, Wools-Kaloustian K, Brazier E, Althoff KN, Jaquet A, Duda SN, et al. Global trends in CD4 count measurement and distribution at first antiretroviral treatment initiation. Clin Infect Dis. 2024 Nov 6;ciae548.

54. Musengimana G, Umugisha JP, Habinshuti P, Anderson T, Mukesharurema G, Remera E, et al. Characteristics and clinical outcomes of patients presenting with advanced HIV disease in the "treat all" era: a retrospective cohort study from rural Rwanda. BMC Infect Dis. 2022 Aug 25;22(1):706.

55. WHO. Guidelines for Managing Advanced HIV Disease and Rapid Initiation of Antiretroviral Therapy [Internet]. World Health Organization. Geneva, Switzerland; 2017 [cited 2025 Jan 14] p. 56. Available from: https://www.who.int/publications/i/item/9789241550062

56. Takamiya M, Takarinda K, Balachandra S, Musuka G, Radin E, Hakim A, et al. Missed opportunities for TB diagnostic testing among people living with HIV in Zimbabwe: Cross-sectional analysis of the Zimbabwe Population-based HIV Impact Assessment (ZIMPHIA) survey 2015-16. J Clin Tuberc Mycobact Dis. 2024 May;35:100427.

57. Gunde L, Wang A, Payne D, O'Connor S, Kabaghe A, Kalata N, et al. Characteristics of TPT initiation and completion among people living with HIV. IJTLD OPEN. 2024 Jan 1;1(1):11–9.

58. Pease C, Hutton B, Yazdi F, Wolfe D, Hamel C, Quach P, et al. Efficacy and completion rates of rifapentine and isoniazid (3HP) compared to other treatment regimens for latent tuberculosis infection: a systematic review with network meta-analyses. BMC Infect Dis. 2017 Apr 11;17(1):265.

59. Tseng SY, Huang YS, Chang TE, Perng CL, Huang YH. Hepatotoxicity, efficacy and completion rate between 3 months of isoniazid plus rifapentine and 9 months of isoniazid in treating latent tuberculosis infection: A systematic review and meta-analysis. J Chin Med Assoc JCMA. 2021 Nov 1;84(11):993–1000.

60. Lawn SD, Badri M, Wood R. Tuberculosis among HIV-infected patients receiving HAART: long term incidence and risk factors in a South African cohort. AIDS. 2005 Dec 2;19(18):2109–16.

61. Mellors JW, Muñoz A, Giorgi JV, Margolick JB, Tassoni CJ, Gupta P, et al. Plasma viral load and CD4+ lymphocytes as prognostic markers of HIV-1 infection. Ann Intern Med. 1997 Jun 15;126(12):946–54.

62. United Nations, Department of Economic and Social Affairs, Population Division. Population size [Internet]. World Population Prospects; 2019 [cited 2025 Mar 18]. Available from: https://population.un.org/wpp/Download/Standard/CSV/

63. World Health Organization. Disease burden and mortality estimates: cause-specific mortality, 2000-2016 [Internet]. [cited 2023 Jun 23]. Available from: https://www.who.int/healthinfo/global_burden_disease/estimates/en/

64. Preston SH, Heuveline P, Guillot M. Demography: Measuring and modeling population processes. Oxford: Blackwell Publishers; 2001.

65. Venter WDF, Moorhouse M, Sokhela S, Fairlie L, Mashabane N, Masenya M, et al. Dolutegravir plus two different prodrugs of tenofovir to treat HIV. N Engl J Med. 2019 Aug 29;381(9):803–15.

66. NAMSAL ANRS 12313 Study Group, Kouanfack C, Mpoudi-Etame M, Omgba Bassega P, Eymard-Duvernay S, Leroy S, et al. Dolutegravir-based or low-dose efavirenz-based regimen for the treatment of HIV-1. N Engl J Med. 2019 Aug 29;381(9):816–26.

67. Sokhela S, Venter WDF, Bosch B, Woods J, McCann K, Akpomiemie G, et al. Final 192-Week Efficacy and Safety Results of the ADVANCE Trial, Comparing 3 First-line Antiretroviral Regimens. Open Forum Infect Dis. 2024 Mar;11(3):ofae007.

68. Cheng Y, Sauer B, Zhang Y, Nickman NA, Jamjian C, Stevens V, et al. Adherence and virologic outcomes among treatment-naïve veteran patients with human immunodeficiency virus type 1 infection. Medicine (Baltimore). 2018 Jan;97(2):e9430.

69. Aboud M, Kaplan R, Lombaard J, Zhang F, Hidalgo JA, Mamedova E, et al. Dolutegravir versus ritonavir-boosted lopinavir both with dual nucleoside reverse transcriptase inhibitor therapy in adults with HIV-1 infection in whom first-line therapy has failed (DAWNING): an open-label, non-inferiority, phase 3b trial. Lancet Infect Dis. 2019 Mar;19(3):253–64.

70. Paton NI, Kityo C, Hoppe A, Reid A, Kambugu A, Lugemwa A, et al. Assessment of second-line antiretroviral regimens for HIV therapy in Africa. N Engl J Med. 2014 Jul 17;371(3):234–47.

71. Walmsley SL, Antela A, Clumeck N, Duiculescu D, Eberhard A, Gutiérrez F, et al. Dolutegravir plus abacavir–lamivudine for the treatment of HIV-1 infection. N Engl J Med. 2013 Nov 7;369(19):1807–18.

72. Gachara G, Mavhandu LG, Rogawski ET, Manhaeve C, Bessong PO. Evaluating adherence to antiretroviral therapy using pharmacy refill records in a rural treatment site in south africa. AIDS Res Treat. 2017 Jan 31;2017:e5456219.

73. Haas AD, Zaniewski E, Anderegg N, Ford N, Fox MP, Vinikoor M, et al. Retention and mortality on antiretroviral therapy in sub-Saharan Africa: collaborative analyses of HIV treatment programmes. J Int AIDS Soc. 2018 Feb;21(2).

74. Ambia J, Kabudula C, Risher K, Xavier Gómez-Olivé F, Rice BD, Etoori D, et al. Outcomes of patients lost to follow-up after antiretroviral therapy initiation in rural north-eastern South Africa. Trop Med Int Health TM IH. 2019 Jun;24(6):747–56.

75. Broger T, Nicol MP, Székely R, Bjerrum S, Sossen B, Schutz C, et al. Diagnostic accuracy of a novel tuberculosis point-of-care urine lipoarabinomannan assay for people living with HIV: A meta-analysis of individual in- and outpatient data. PLoS Med. 2020 May;17(5):e1003113.

76. Field N, Lim MS, Murray J, Dowdeswell RJ, Glynn JR, Sonnenberg P. Timing, rates, and causes of death in a large South African tuberculosis programme. BMC Infect Dis. 2014 Dec 21;14:3858.

77. Ross JM, Badje A, Rangaka MX, Walker AS, Shapiro AE, Thomas KK, et al. Isoniazid Preventive Therapy Added to ART to Prevent TB: An Individual Participant Data Meta-Analysis. Lancet HIV. 2021 Jan 1;8(1):e8–15.

78. Jarvis JN, Lawrence DS, Meya DB, Kagimu E, Kasibante J, Mpoza E, et al. Single-dose liposomal amphotericin B treatment for cryptococcal meningitis. N Engl J Med. 2022 Mar 24;386(12):1109–20.

79. Anglaret X, Chêne G, Attia A, Toure S, Lafont S, Combe P, et al. Early chemoprophylaxis with trimethoprim-sulphamethoxazole for HIV-1-infected adults in Abidjan, Côte d'Ivoire: a randomised trial. The Lancet. 1999 May 1;353(9163):1463–8.

80. Yazdanpanah Y, Losina E, Anglaret X, Goldie SJ, Walensky RP, Weinstein MC, et al. Clinical impact and cost-effectiveness of co-trimoxazole prophylaxis in patients with HIV/AIDS in Côte d'Ivoire: a trial-based analysis. AIDS. 2005 Aug 12;19(12):1299–308.

81. Suthar AB, Vitoria MA, Nagata JM, Anglaret X, Mbori-Ngacha D, Sued O, et al. Co-trimoxazole prophylaxis in adults, including pregnant women, with HIV: a systematic review and meta-analysis. Lancet HIV. 2015 Apr;2(4):e137–50.

82. Walker A, Ford D, Gilks C, Munderi P, Ssali F, Reid A, et al. Daily co-trimoxazole prophylaxis in severely immunosuppressed HIV-infected adults in Africa started on combination antiretroviral therapy: an observational analysis of the DART cohort. Lancet. 2010 Apr 10;375(9722):1278–86.

83. Sanders GD, Neumann PJ, Basu A, Brock DW, Feeny D, Krahn M, et al. Recommendations for conduct, methodological practices, and reporting of cost-effectiveness analyses: second panel on cost-effectiveness in health and medicine. JAMA. 2016 Sep 13;316(10):1093–103.

84. Population, total - Malawi [Internet]. [cited 2025 Mar 18]. Available from: https://data.worldbank.org/indicator/SP.POP.TOTL?locations=MW

SUPPLEMENTARY TABLES AND FIGURES

Table S1. Detailed input parameters for HIV natural history.

Parameter	Value	Reference
Initial plasma HIV-1 RNA distribution, copies/mL, %		
>100,000	42	
30,000-100,000	28	
10,000-30,000	18	(60)
3,000-10,000	8	
500-3,000	2	
<500	1	
Monthly CD4 decline in cells/µL stratified by plasma HIV-1 RNA among PLWH not taking ART, copies/mL, mean (SD)		
>100,000	6.4 (0.3)	
30,000-100,000	6.4 (0.3)	
10,000-30,000	5.4 (0.2)	(61)
3,000-10,000	4.6 (0.2)	
500-3,000	3.7 (0.2)	
<500	3.0 (0.3)	
Probability of non-AIDS-related mortality (range by age and sex), monthly, %		
15-24 years	0.007-0.015	
25-39 years	0.012-0.024	
40-49 years	0.022-0.049	(62–64)
50-59 years	0.044-0.113	
≥ 60 years	0.105-2.416	
Probability of HIV-related mortality (stratified by CD4 and history of prior OI), monthly, %		
Off ART	0.156-9.530	(29)
No/Mild OI History		
CD4 ≥ 500	0.156	
350 ≤ CD4 < 500	0.252	
200 ≤ CD4 < 350	0.407	
100 ≤ CD4 < 200	1.294	
50 ≤ CD4 < 100	2.140	
CD4 < 50	4.019	
Severe OI History		
CD4 ≥ 500	0.156	
350 ≤ CD4 < 500	0.252	
200 ≤ CD4 < 350	0.407	
100 ≤ CD4 < 200	3.253	
50 ≤ CD4 < 100	5.075	
CD4 < 50	9.530	
On ART	0.006-4.407	(29,37)
No/Mild OI History		
CD4 ≥ 500	0.006	
350 ≤ CD4 < 500	0.010	
200 ≤ CD4 < 350	0.016	
100 ≤ CD4 < 200	0.117	
50 ≤ CD4 < 100	0.195	
CD4 < 50	1.829	
Severe OI History		
CD4 ≥ 500	0.006	
350 ≤ CD4 < 500	0.010	
200 ≤ CD4 < 350	0.016	
100 ≤ CD4 < 200	0.297	
50 ≤ CD4 < 100	0.468	
CD4 < 50	4.407	

Abbreviations: AIDS, acquired immunodeficiency syndrome; ART, antiretroviral therapy; CD4, cluster of differentiation 4; HIV, human immunodeficiency virus; OI, opportunistic infection; PLWH, people living with HIV; RNA, ribonucleic acid; SD, standard deviation.

Table S2. Detailed input parameters for HIV treatment and care continuum.

Parameter		Value	Reference
HIV treatment characteristics			
TDF/FTC+DTG (first-line ART)			
HIV-1 RNA suppression at 6 months, %		92	Derived from (65–67)
Virologic nonsuppression after suppressing during the first 6 months, monthly, %		0.2	Derived from (65–67)
AZT/3TC+LPV/r (second-line ART)			
HIV-1 RNA suppression at 6 months, %		73	(68–70)
Virologic nonsuppression after suppressing during the first 6 months, monthly, %		0.2	(70)
CD4 increase for all ART lines, mean (SD), monthly, cells/µL			
	Sub-cohort with CD4 <200/µL	Sub-cohort with CD4 ≥ 200/µL	
≤1 month	79 (30)	107 (30)	Calibrated to (14,71)
1-3 months	21 (2)	5 (2)	
>3 months	7 (2)	5 (2)	
Adherence			
ART adherence, mean (SD), %[a]		93 (5.3)	(72)
Engagement in care			
Probability of loss to follow-up, annual, %		8.5	Calibrated to (73)
Probability of return to care after 12 months, monthly, %		1.3	(74)
Probability of return to care after OI, one-time, %		50	Assumption

Abbreviations: HIV, human immunodeficiency virus; TDF/FTC+DTG, tenofovir disoproxil fumarate and emtricitabine with dolutegravir; ART, antiretroviral therapy; RNA, ribonucleic acid; AZT/3TC+LPV/r, zidovudine and lamivudine with lopinavir/ritonavir; CD4, cluster of differentiation 4; SD, standard deviation; OI, opportunistic infection; TB, tuberculosis.

[a] Measured as pill counts, MEMS-Caps, Wise-pills, pharmacy refills, etc.

Table S3. Detailed input parameters for natural history, diagnosis, treatment, and prevention of TB.

Parameter	Value			Reference
**Cohort characteristics at model entry**				
Prevalence in the starting population, %				
Active TB disease				
CD4 strata, /µL	Sub-cohort with WHO stage 3/4 disease	Sub-cohort without WHO stage 3/4 disease	Full cohort	
>350	14	6	10	
200 – 350	16	7	12	
100 – 200	27	7	18	(8,9,11)
50 – 100	40	9	28	
< 50	51	13	37	
Latent TB infection				
CD4 strata, /µL	Sub-cohort with WHO stage 3/4 disease	Sub-cohort without WHO stage 3/4 disease	Full cohort	
>350	43	51	47	
200 – 350	41	50	45	
100 – 200	30	50	39	(7–9)
50 – 100	17	48	29	
< 50	6	44	20	
No TB infection		43		(7)
Prevalence of 1 or more symptoms consistent with active TB [a] at model start, %				
Active TB				
CD4 strata, /µL	Sub-cohort with WHO stage 3/4 disease	Sub-cohort without WHO stage 3/4 disease	Full cohort	
> 200	100	0	71	(11)
≤ 200	100	0	87	
Latent TB infection or no TB infection				
CD4 strata, /µL		Full cohort		
> 200		51		(11)
≤ 200		67		
	Drug-susceptible TB		Drug-resistant TB	
Prevalence of TB strains, %	99		1	(8)
**Natural history**				
Probability of incident TB infection, monthly %				
Age, years				
<18		0.34		
18-25		0.56		(12)
26-45		0.80		
>45		0.68		
Progression from latent TB infection to active TB disease, monthly, %				
CD4 strata, /µL				
>500		0.24		
350 – 500		0.45		
200 – 350		0.51		(13)
100 – 200		1.28		
< 100		3.19		
Monthly probability of mortality from active TB if untreated or failed TB treatment, %		7		Calibrated to (14)
**TB diagnostics**				
Probability of TB test being accessed by eligible PLWH, [b] %		Sputum Xpert<br>79	Urine LAM<br>91	(15,16)
Sample provision by PLWH, %		Sputum<br>87	Urine<br>100	(4) , Assumption
Diagnostic yield in outpatients presenting with TB symptom(s), %				
CD4 strata, /µL		Xpert	Xpert + LAM	
>200		70	72	
100 – 200		68	73	(4)
< 100		68	85	
Specificity, %		98	95	(4,75)
**Empiric TB treatment**				
Probability of PLWH eligible for empiric TB treatment, %		PLWH with active TB	PLWH without active TB	
If Xpert/LAM is not available (*ART only*)		30	12	(8)
If patient does not accept Xpert/LAM, or receives a negative test result		17	7	

Parameter	Value	Reference
Treatment		
Probability of initiating TB treatment, among PLWH who receive a TB diagnosis, %	91	(17)
RHZE (Rifampin, Isoniazid, Pyrazinamide, Ethambutol) regimen		
Duration, months	6	
Efficacy among PLWH who complete the full treatment course, %		
PLWH with drug-susceptible TB	98	(18)
PLWH with drug-resistant TB	69	
Overall probability of treatment regimen completion among PLWH who initiate it, %	88	(17)
Minor toxicity probability, %	1	Assumption
Major toxicity probability, %	0.55	(19)
Probability of death from major toxicity, %	2.1	(76)
TB prophylaxis		
TB preventive therapy (TPT) regimen using Isoniazid		
Duration of regimen, months	6	(55)
Efficacy against TB infection and reactivation, %	43	(13,77)
Duration of efficacy after completing TPT course, months	24	(13)
Probability of initiating TPT regimen among eligible PLWH, %	79 [d]	
Probability of TPT regimen completion among PLWH who initiate TPT, %	87	(21)
Probability of developing INH resistance if active TB patients are incorrectly given TPT, %	50	(5)

Abbreviations: TB, tuberculosis; CD4, cluster of differentiation 4; WHO, World Health Organization; LAM, lateral flow lipoarabinomannan; PLWH, people living with HIV; TPT, TB preventive therapy; INH, isoniazid.

[a] WHO TB symptoms include: cough, fever, weight loss, and night sweats

[b] Patients are eligible for TB test if they have 1 or more WHO TB symptoms

[d] Maphosa T, EGPAF, unpublished

Table S4. Detailed input parameters for the natural history, diagnosis, treatment, and prevention of cryptococcal infection.

Parameter	Value	Reference
Cryptococcal infection natural history		
Initial asymptomatic cryptococcemia prevalence by CD4 count, /µL, %		
>200	0	
100-200	2.0	(23)
<100	6.7	
Monthly cryptococcal infection incidence by CD4 count, /µL, %		
>200	0	
100-200	0.13	(22,23)
<100	0.44	
Monthly probability of progression from asymptomatic cryptococcemia to symptomatic meningitis from time since infection, %		
≤6 months	5.33	(24,78)
>6 months	0	
Probability of asymptomatic cryptococcal infection-related mortality, monthly, %	0	Assumption
Probability of untreated symptomatic cryptococcal meningitis-related mortality, monthly, %	78	(26)
Asymptomatic cryptococcemia diagnostics		
Serum CrAg LFA		
Sensitivity, %	97.6	(6)
Specificity, %	98.1	
Probability of test uptake by eligible PLWH, %	75	Assumption
Probability of initiating preemptive fluconazole, among PLWH with a positive test result, %	90	Assumption
Symptomatic meningitis diagnostics		
LP with CSF CrAg LFA, among symptomatic PLWH		
Sensitivity, %	98.9	(28)
Specificity, %	98.9	
Probability of having access to an LP, among symptomatic PLWH, %	90	Assumption
Probability of accepting the test, among PLWH who can access it, %	73	(28)
Probability of initiating cryptococcal meningitis treatment, among PLWH with a positive test result, %	98	Assumption
Probability of initiating meningitis treatment empirically, if symptomatic and LP not obtained, %	88	Assumption
Preemptive fluconazole characteristics		
Efficacy against infection and reinfection, %	100	Assumption
Efficacy against progression from asymptomatic cryptococcal antigenemia to symptomatic meningitis, %	72	(25)
Cryptococcal meningitis treatment characteristics		
Efficacy against infection and reinfection, %	100	Assumption
Efficacy against progression from asymptomatic cryptococcemia to symptomatic meningitis, %	100	Assumption
Mortality reduction on treatment, stratified by number of months of treatment completed, %		
≤1 month	89	(78)
1-3 months	98	
>3 months	100	

Abbreviations: CD4, cluster of differentiation 4; CrAg, cryptococcal antigen; LFA, lateral flow assay; PLWH, people living with HIV; LP, lumbar puncture; CSF, cerebrospinal fluid.

Table S5. Detailed input parameters for the natural history, diagnosis, treatment, and prevention of severe malaria, serious bacterial infections, and other WHO stage 3/4 diseases.

Parameter	Value	Reference
Monthly probability of OI incidence (stratified by CD4), off ART, %		
Off ART		
Severe malaria	0.024	(33–35)
Severe bacterial infections	0.042-3.677	(14,29–31)
CD4 ≥ 500	0.042	
350 ≤ CD4 < 500	0.457	
200 ≤ CD4 < 350	0.549	
100 ≤ CD4 < 200	1.147	
50 ≤ CD4 < 100	2.194	
CD4 < 50	3.677	
Other WHO stage 3 or 4 diseases		(14,29)
CD4 ≥ 500	0.249	
350 ≤ CD4 < 500	0.290	
200 ≤ CD4 < 350	0.526	
100 ≤ CD4 < 200	0.900	
50 ≤ CD4 < 100	1.734	
CD4 < 50	4.588	
On ART		
Severe malaria	0.017	
CD4 ≥ 500	0.017	
350 ≤ CD4 < 500	0.017	
200 ≤ CD4 < 350	0.017	
100 ≤ CD4 < 200	0.017	
50 ≤ CD4 < 100	0.017	
CD4 < 50	0.017	
Severe bacterial infections	0.028-2.515	
CD4 ≥ 500	0.028	
350 ≤ CD4 < 500	0.311	
200 ≤ CD4 < 350	0.374	(14,37)
100 ≤ CD4 < 200	0.781	
50 ≤ CD4 < 100	1.497	
CD4 < 50	2.515	
Other WHO stage 3 or 4 diseases		
CD4 ≥ 500	0.169	
350 ≤ CD4 < 500	0.197	
200 ≤ CD4 < 350	0.358	
100 ≤ CD4 < 200	0.613	
50 ≤ CD4 < 100	1.182	
CD4 < 50	3.143	
OI mortality probability in month of incident OI, %		
Severe malaria	28.1	(33)
Severe bacterial infections	30.0	(14)
Other WHO stage 3 or 4 diseases	18.7	(14)
Co-trimoxazole prophylaxis characteristics		
Efficacy in preventing incident OIs, %		
Severe malaria	88.4	
Severe bacterial infections	49.8	(79,80)
Other WHO stage 3 or 4 diseases	15.0	(81,82)
Minor toxicity probability, %	16.7	(80)
Major toxicity probability, %	6.5	(80)

Abbreviations: WHO, World Health Organization; OI, opportunistic infection; CD4, cluster of differentiation 4; ART, antiretroviral therapy.

Table S6. Detailed costs parameters (USD 2023).

Parameter	Value	Reference
HIV clinical care costs		
TDF/3TC+DTG, monthly	3.60	(42,47,48)
AZT/3TC+ LPV/r, monthly	18.57	(43)
CD4 count, per test	5.80	(46)
HVL test, per test	19.58	(48)
Routine care costs by CD4 count, /µL, monthly		
CD4 ≥ 500	2.86	
350 ≤ CD4 < 500	3.96	
200 ≤ CD4 < 350	4.90	
100 ≤ CD4 < 200	10.77	(29,40,51)
50 ≤ CD4 < 100	18.51	
CD4 < 50	26.24	
Death cost, any cause of death	104.26	
TB diagnostics, treatment, and prophylaxis costs		
Sputum Xpert, per test	15.72	(48)
Urine LAM, per test	5.69	(42)
Chest X-ray, per test	8.59	(15)
Sputum smear microscopy, per test	8.07	(40)
TPT, monthly	0.95	(42)
TB treatment initiation, one time [a]		(29,40,51)
CD4 <200/µL with or without WHO stage 3/4 disease	41.22	(40)
CD4 >=200/µL with WHO stage 3/4 disease	20.61	(40)
RHZE treatment, months 1-2	11.75	(44)
RHZE treatment, months 3-6	5.97	(44)
Cryptococcal infection costs		
Serum CrAg LFA	3.77	(42)
LP with CSF CrAg LFA	19.77	(50)
Fluconazole preemptive therapy, average monthly	4.90	(42)
CM medication costs		
Fluconazole, per 200mg pill	0.08	
Flucytosine, per 500mg pill	0.94	(42)
Amphotericin B liposomal, per 50mg vial	20.31	
Other treatment costs		
CM hospitalization, per hospital bed day	41.42	(40)
Laboratory monitoring, per day	3.88	(15)
OI treatment and prophylaxis costs		
OI treatment costs, per incident OI		
Malaria	185.49	
Severe bacterial infections	142.80	(29,40,51)
Other WHO clinical stage 3 or 4 diseases	86.72	
OI prophylaxis cost		
Co-trimoxazole prophylaxis, monthly	0.97	(42)
Drug toxicity costs		
Minor toxicity [b], one time	1.10	(15,40)
Major toxicity [c], one time	189.62	

Abbreviations: USD, United States dollar; HIV, human immunodeficiency virus; TDF/3TC+DTG, tenofovir disoproxil fumarate and lamivudine with dolutegravir; AZT/3TC+LPV/r, zidovudine and lamivudine with lopinavir/ritonavir; HVL, HIV viral load; LAM, lipoarabinomannan assay; TPT, TB preventive therapy; RHZE, Rifampin, Isoniazid, Pyrazinamide, Ethambutol; CrAg, cryptococcal antigen; LFA, lateral flow assay; LP, lumbar puncture; CSF, cerebrospinal fluid; CM, cryptococcal meningitis; OI, opportunistic infection; WHO, World Health Organization.

[a] This includes costs (apart from diagnostics and medication) associated with starting TB treatment, like additional clinic visits, supplies, hospitalization, etc.

[b] We assume that minor toxicity would require an outpatient visit (~20 minutes of nurse time).

[c] We assume that major toxicity would require about one week of inpatient care.

Table S7. Detailed quality of life model inputs and multipliers for opportunistic infections and CD4 counts in PLWH.

Parameter	Value	Reference
Opportunistic infection	QoL input	
Tuberculosis	0.62	
Cryptococcal meningitis	0.48	
Severe bacterial infection	0.54	(40)
Malaria	0.52	
Other opportunistic infections	0.50	
Toxicity	QoL input	
Major toxicity	0.75	(40)

Abbreviations: PLWH, persons living with HIV; QoL, quality of life

Table S8. Cohort characteristics among PLWH initiating standard care in REALITY trial validation (14)

Parameter	Value
Age, years, mean (SD)	36 (9)
Female at birth, %	46
CD4 count, mean (SD), /µL	36 (33)
Loss to follow-up, annual, %	3.5
Diagnosed with active TB, %	15
Diagnosed with cryptococcal meningitis, %	1.3

Abbreviations: PLWH, people living with HIV; REALITY, Reduction of Early Mortality in HIV-Infected Adults and Children Starting Antiretroviral Therapy trial; SD, standard deviation; TB, tuberculosis.

Table S9. Model-projected cost-effectiveness results for the WHO-recommended AHD package of care in univariate sensitivity analysis.

Parameter	Disc. QALYs [a]	Disc. lifetime costs ($) [ab]	ICER ($/QALY) [b]
**Clinical parameters**			
TB			
Additional diagnostic yield of LAM, [c] %			
Lower bound: 1+	12.21	1,740	580
Upper bound: 29+	12.35	1,760	570
TPT efficacy in preventing TB reactivation, %			
Lower bound: 5	12.18	1,740	570 [d]
Upper bound: 45	12.26	1,750	580
Cryptococcal infection			
Probability of linkage to preemptive fluconazole, %			
Lower bound: 25	12.24	1,750	560 [e,f]
Upper bound: 100	12.24	1,750	560
Cryptococcemia prevalence at model start, %			
Lower bound: 0.5	12.25	1,740	560 [e]
Upper bound: 23	12.23	1,750	560
Preemptive fluconazole efficacy against progression to meningitis, %			
Lower bound: 20	12.24	1,750	690 [e,f]
Upper bound: 100	12.24	1,750	560
Bacterial infections			
Monthly SBI incidence, %			
Lower bound: 0.67	12.19	1,610	300 [g]
Upper bound: 2.69	11.93	1,720	430
Mortality from SBI, %			
Lower bound: 5	12.44	1,650	300 [g]
Upper bound: 50	11.97	1,710	460
CTX efficacy against SBIs, %			
Lower bound: 11	11.96	1,590	310 [g]
Upper bound: 69	12.42	1,760	390
Cohort characteristics			
Cohort proportion with AHD, %			
Lower bound: 5	12.56	1,620	350 [g]
Upper bound: 50	11.30	1,700	500
**Cost parameters (2023 USD)**			
TB			
LAM cost, per test			
Lower bound: 13.64	12.24	1,740	580
Upper bound: 21.93	12.24	1,770	580
TPT cost, monthly			
Lower bound: 0.48	12.24	1,740	580
Upper bound: 2.00	12.24	1,750	580
Cryptococcal infection			
CrAg cost, per test			
Lower bound: 1.89	12.24	1,750	560
Upper bound: 7.92	12.24	1,750	560
Preemptive fluconazole therapy cost, monthly			
Lower bound: .79	12.24	1,750	560
Upper bound: 7.9	12.24	1,750	570 [e,f]
Bacterial infections			
CTX cost, monthly			
Lower bound: 0.29	12.24	1,660	310
Upper bound: 2.59	11.96	1,590	310 [e,f]

Abbreviations: WHO, World Health Organization; AHD, advanced HIV disease; ICER, incremental cost effectiveness ratio; QALY, quality-adjusted life years; TB, tuberculosis; LAM, lipoarabinomannan; TPT, TB preventive therapy; SBI, serious bacterial infection; CTX, co-trimoxazole; USD, US dollars; CrAg, cryptococcal antigen.

[a] Discounted at 3% per year.

[b] Total costs and ICERs are rounded to the nearest $10.

[c] Additional TB cases diagnosed when LAM is used (in addition to Xpert).

[d] The WHO-recommended AHD package is dominated by ART+Xpert+LAM+CTX+CrAg; the cost-effective strategy in this case is *ART+Xpert+LAM+CTX+CrAg (No TPT)*.

[e] The WHO-recommended AHD package is not cost-effective here (i.e., <$600/QALY); the cost-effective strategy in this case is *ART+Xpert+LAM+CTX+TPT (No CrAg)*.
[f] The WHO-recommended AHD package is dominated by *ART+Xpert+LAM+CTX+TPT (No CrAg)*.
[g] The WHO-recommended AHD package is not cost-effective here (i.e., <$600/QALY); the cost-effective strategy in this case is *ART+Xpert+LAM+CrAg+TPT (No CTX)*.

Table S10. Model-projected clinical outcomes, costs, and cost-effectiveness of different strategies for the prevention, diagnosis, and treatment of AHD among PLWH in Malawi who experience lower uptake of AHD package in absence of a CD4 count.

Availability of CD4	Strategy	1y survival (%) [a]	Undisc. QALYs	Disc. QALYs [b]	Disc. lifetime costs ($) [bc]	ICER ($/QALY) [cd]
No	*ART only*	91.55	17.45	11.28	1,440	--
No	***ART+Xpert***	**92.49**	**18.01**	**11.59**	**1,500**	**180**
No	*ART+Xpert+LAM*	92.63	18.15	11.65	1,510	dom
No	*ART+Xpert+LAM+TPT*	92.69	18.19	11.68	1,520	dom
No	*ART+Xpert+CTX*	92.87	18.39	11.78	1,610	DOM
No	*ART+Xpert+LAM+CTX*	93.01	18.53	11.85	1,620	DOM
No	*ART+Xpert+LAM+CTX+TPT*	93.12	18.60	11.89	1,630	DOM
Yes	*ART only*	91.56	17.45	11.28	1,450	DOM
Yes	*ART+Xpert*	92.74	18.48	11.81	1,540	dom
**Yes**	***ART+Xpert+CrAg***	**92.75**	**18.48**	**11.81**	**1,540**	**200**
Yes	*ART+Xpert+LAM*	92.88	18.67	11.90	1,580	dom
Yes	*ART+Xpert+LAM+CrAg*	92.89	18.67	11.90	1,580	dom
Yes	*ART+Xpert+LAM+TPT*	93.02	18.76	11.96	1,590	dom
**Yes**	***ART+Xpert+LAM+CrAg+TPT***	**93.05**	**18.76**	**11.96**	**1,590**	**310**
Yes	*ART+Xpert+CTX*	93.23	19.01	12.09	1,700	dom
Yes	*ART+Xpert+CTX+CrAg*	93.24	19.02	12.09	1,700	dom
Yes	*ART+Xpert+LAM+CTX*	93.36	19.21	12.18	1,740	dom
Yes	*ART+Xpert+LAM+CTX+CrAg*	93.38	19.22	12.18	1,740	dom
Yes	*ART+Xpert+LAM+CTX+TPT*	93.52	19.31	12.24	1,750	DOM
Yes	***WHO-recommended AHD Package***	**93.54**	**19.30**	**12.24**	**1,750**	**580**

Abbreviations: AHD, advanced HIV disease; PLWH; people living with HIV; CD4, cluster of differentiation 4; Undisc., undiscounted; Disc., discounted; QALY, quality-adjusted life year; ICER, incremental cost-effectiveness ratio; ART, antiretroviral therapy; LAM, lateral flow lipoarabinomannan; CrAg, cryptococcal antigen; TPT, TB preventive therapy; CTX, co-trimoxazole.

[a] The percentage of the cohort that survives 1 year in the model.

[b] Discounted at 3% per year.

[c] Total costs and ICERs are rounded to the nearest $10.

[d] Strategies are "weakly dominated" (dom) if another intervention provides a greater increase in QALYs for the same cost and "strongly dominated" (DOM) if another intervention provides a greater increase in QALYs for a lower cost (i.e., the ICER is smaller). We report undiscounted health outcomes but use discounted cost outcomes to calculate ICERs, as recommended by the Second Panel on Cost-Effectiveness in Health and Medicine (83).

Table S11. Model-projected clinical outcomes, costs, and cost-effectiveness of different strategies for the prevention, diagnosis, and treatment of AHD among PLWH in Malawi who experience a one-month delay in ART initiation in absence of a CD4 count.

Availability of CD4	Strategy	1y survival (%) [a]	Undisc. QALYs	Disc. QALYs [b]	Disc. lifetime costs ($) [bc]	ICER ($/QALY) [cd]
No	*ART only*	89.64	16.90	10.94	1,410	--
No	*ART+Xpert*	90.81	17.91	11.46	1,500	dom
No	*ART+Xpert+LAM*	90.96	18.09	11.55	1,530	dom
No	*ART+Xpert+LAM+TPT*	91.17	18.20	11.61	1,540	dom
No	*ART+Xpert+CTX*	91.49	18.47	11.75	1,650	DOM
No	*ART+Xpert+LAM+CTX*	91.64	18.66	11.84	1,690	DOM
No	*ART+Xpert+LAM+CTX+TPT*	91.82	18.76	11.90	1,700	DOM
**Yes**	***ART only***	**91.56**	**17.45**	**11.28**	**1,450**	**130**
Yes	*ART+Xpert*	92.74	18.48	11.81	1,540	dom
Yes	*ART+Xpert+CrAg*	92.75	18.48	11.81	1,540	170
Yes	*ART+Xpert+LAM*	92.88	18.67	11.90	1,580	dom
Yes	*ART+Xpert+LAM+CrAg*	92.89	18.67	11.90	1,580	dom
Yes	*ART+Xpert+LAM+TPT*	93.02	18.76	11.96	1,590	dom
**Yes**	***ART+Xpert+LAM+CrAg+TPT***	**93.05**	**18.76**	**11.96**	**1,590**	**310**
Yes	*ART+Xpert+CTX*	93.23	19.01	12.09	1,700	dom
Yes	*ART+Xpert+CTX+CrAg*	93.24	19.02	12.09	1,700	dom
Yes	*ART+Xpert+LAM+CTX*	93.36	19.21	12.18	1,740	dom
Yes	*ART+Xpert+LAM+CTX+CrAg*	93.38	19.22	12.18	1,740	dom
Yes	*ART+Xpert+LAM+CTX+TPT*	93.52	19.31	12.24	1,750	DOM
**Yes**	***WHO-recommended AHD Package***	**93.54**	**19.30**	**12.24**	**1,750**	**580**

Abbreviations: AHD, advanced HIV disease; PLWH; people living with HIV; ART, antiretroviral therapy; CD4, cluster of differentiation 4; Undisc., undiscounted; Disc., discounted; QALY, quality-adjusted life year; ICER, incremental cost-effectiveness ratio; LAM, lateral flow lipoarabinomannan; CrAg, cryptococcal antigen; TPT, TB preventive therapy; CTX, co-trimoxazole.

[a] The percentage of the cohort that survives 1 year in the model.

[b] Discounted at 3% per year.

[c] Total costs and ICERs are rounded to the nearest $10.

[d] Strategies are "weakly dominated" (dom) if another intervention provides a greater increase in QALYs for the same cost and "strongly dominated" (DOM) if another intervention provides a greater increase in QALYs for a lower cost (i.e., the ICER is smaller). We report undiscounted health outcomes but use discounted cost outcomes to calculate ICERs, as recommended by the Second Panel on Cost-Effectiveness in Health and Medicine (83).

SUPPLEMENTARY FIGURE LEGENDS

Figure S1. Schematic of the elements in the *WHO-recommended AHD Package*.
Some or all these elements are included in the 13 strategies.
Abbreviations**:** CD4, cluster of differentiation 4; PLWH, people living with HIV; ART, antiretroviral therapy; TB, tuberculosis; LAM, lipoarabinomannan; TPT, TB preventive therapy; CrAg, cryptococcal antigen; CTX, co-trimoxazole; AHD, advanced HIV disease.

Figure S2. Overview of TB module health states and transitions.
A schematic representing the tuberculosis health states and transitions in the CEPAC-I model. Each oval represents a distinct health state. Arrows represent all the possible state transitions; labels in the dashed text boxes show what phenomenon each arrow represents.
Abbreviations**:** Tx, treatment; LTFU, loss to follow up.

Figure S3. Overview of the cryptococcus module health states and transitions.
A schematic representing the cryptococcus health states and transitions in the CEPAC model. Each oval represents a distinct health state. Arrows represent all the possible state transitions; labels in the dashed text boxes show what phenomenon each arrow represents.
Abbreviations**:** Tx, treatment; LTFU, loss to follow up.

Figure S4. **CEPAC-I projections of mortality compared with observed mortality in the REALITY trial.**
The graph shows trend lines representing the overall mortality reported in the REALITY trial at 48 weeks (blue) and projected by the CEPAC-I model (red).
Abbreviations**:** REALITY, Reduction of Early Mortality in HIV-Infected Adults and Children Starting Antiretroviral Therapy trial; CEPAC-I, Cost-effectiveness of Preventing AIDS Complications – International model.

Figure S5. CEPAC-I projected cause of death compared with REALITY trial results for people initiating ART with AHD.
Causes of death include tuberculosis, cryptococcus, serious bacterial infections, other WHO stage 3/4 infections, and other causes of death.
Abbreviations**:** TB, tuberculosis; WHO, World Health Organization; REALITY, Reduction of Early Mortality in HIV-Infected Adults and Children Starting Antiretroviral Therapy trial; CEPAC-I, Cost-effectiveness of Preventing AIDS Complications – International model; ART, antiretroviral therapy; AHD, advanced HIV disease.

Figure S6. Model-projected incremental lifetime clinical outcomes and costs for all strategies with CD4 test available.
Cost-effectiveness frontier showing additional QALYs gained, and dollars cost per strategy, with 'ART only' as the comparator strategy. The dotted line joins the non-dominated strategies; all dominated strategies fall below this line.
Abbreviations**:** QALY, quality-adjusted life year; WHO, World Health Organization; AHD, advanced HIV disease; ART, antiretroviral therapy; Xpert, GeneXpert MTB/RIF assay; LAM, lipoarabinomannan; CTX, cotrimoxazole; CrAg, cryptococcal antigen; TPT, TB preventive therapy; USD, US dollars.

Figure S7. Univariate sensitivity analyses for selected clinical and cost parameters over a range of cost-effectiveness thresholds.
Each panel represents a univariate sensitivity analysis for the stated parameter. The horizontal axis shows the range of values for each parameter; the vertical axis shows the range of cost-effectiveness thresholds. The white X in each panel denotes the base case value of the parameter. The preferred strategy for each value is shown by color at different cost-effectiveness thresholds. The *WHO-recommended AHD Package* (green) is the most frequently preferred strategy at cost-effectiveness thresholds of $520/QALY or greater despite wide ranges of influential input parameters.
Abbreviations**:** QALY, quality-adjusted life year; WHO, World Health Organization; AHD, advanced HIV disease; CTX, co-trimoxazole; TPT, TB preventive therapy; CrAg, cryptococcal antigen; LAM, lipoarabinomannan; Xpert, GeneXpert MTB/RIF assay; SBI, serious bacterial infections; CM, cryptococcal meningitis; ART, antiretroviral therapy.

Figure S8. Model-projected incremental lifetime clinical outcomes and costs for all strategies with and without CD4 test available in two scenarios.
Cost-effectiveness frontiers showing additional QALYs gained, and dollars cost per strategy, with 'ART only' as the comparator strategy. Each strategy is represented by a circle if a CD4 count was available and a triangle, otherwise. The dotted line joins the preferred strategies; all dominated strategies fall below this line. Panel A shows the scenario in which PLWH with undetected AHD in the absence of a CD4 count (all strategies plotted with a triangle) experience lower probabilities for the uptake of TB testing, TPT, CTX prophylaxis, and CrAg screening. Panel B shows the scenario in which PLWH with undetected AHD in the absence of a CD4 count face a 1-month delay in ART initiation compared with PLWH with AHD detected by CD4 count.
Abbreviations**:** QALY, quality-adjusted life year; AHD, advanced HIV disease; CD4, cluster of differentiation 4; ART, antiretroviral therapy; Xpert, GeneXpert MTB/RIF assay; LAM, lipoarabinomannan; CTX, co-trimoxazole; CrAg, cryptococcal antigen; TPT, TB preventive therapy; USD, US dollars.

Figure S9. Detailed budget impact analysis for the *WHO-recommended AHD Package* for people living with HIV in Malawi.
Abbreviations**:** USD, US dollars; OI, opportunistic infection; CTX, co-trimoxazole; TPT, TB preventive therapy; TB, tuberculosis; FLU, fluconazole; CM, cryptococcal meningitis; crypto, cryptococcal infection; CD4, cluster of differentiation 4; HIV, human immunodeficiency virus; ART, antiretroviral therapy; CrAg, cryptococcal antigen; LAM, lipoarabinomannan; Xpert, GeneXpert MTB/RIF assay; WHO, World Health Organization; AHD, advanced HIV disease.

(A)

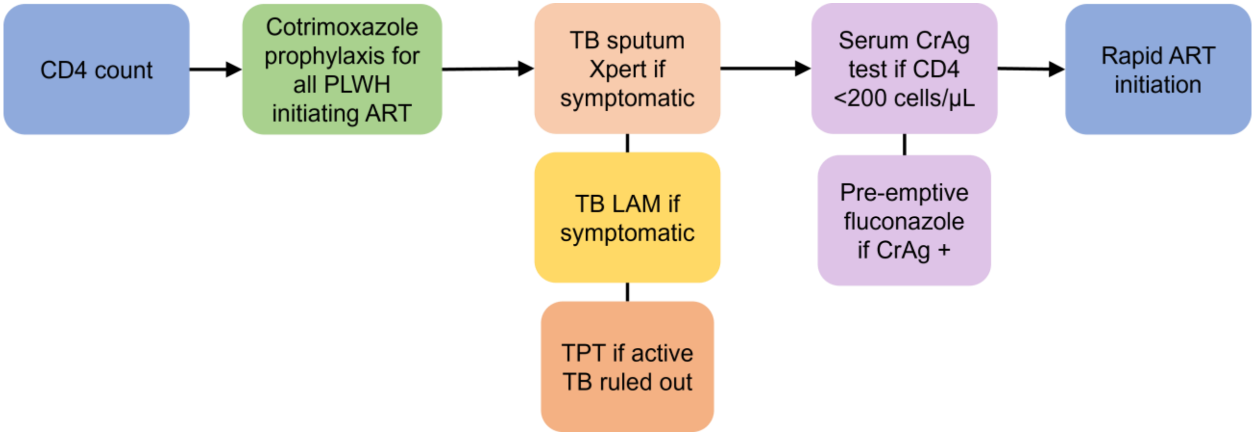

(B)

Strategy #						
1	ART					
2	ART	Xpert				
3	ART	Xpert	LAM			
4	ART	Xpert		CTX		
5	ART	Xpert			CrAg	
6	ART	Xpert	LAM			TPT
7	ART	Xpert	LAM	CTX		
8	ART	Xpert	LAM		CrAg	
9	ART	Xpert		CTX	CrAg	
10	ART	Xpert	LAM	CTX		TPT
11	ART	Xpert	LAM		CrAg	TPT
12	ART	Xpert	LAM	CTX	CrAg	
13	ART	Xpert	LAM	CTX	CrAg	TPT

Figure S1. Schematic of the elements in the *WHO-recommended AHD Package*.

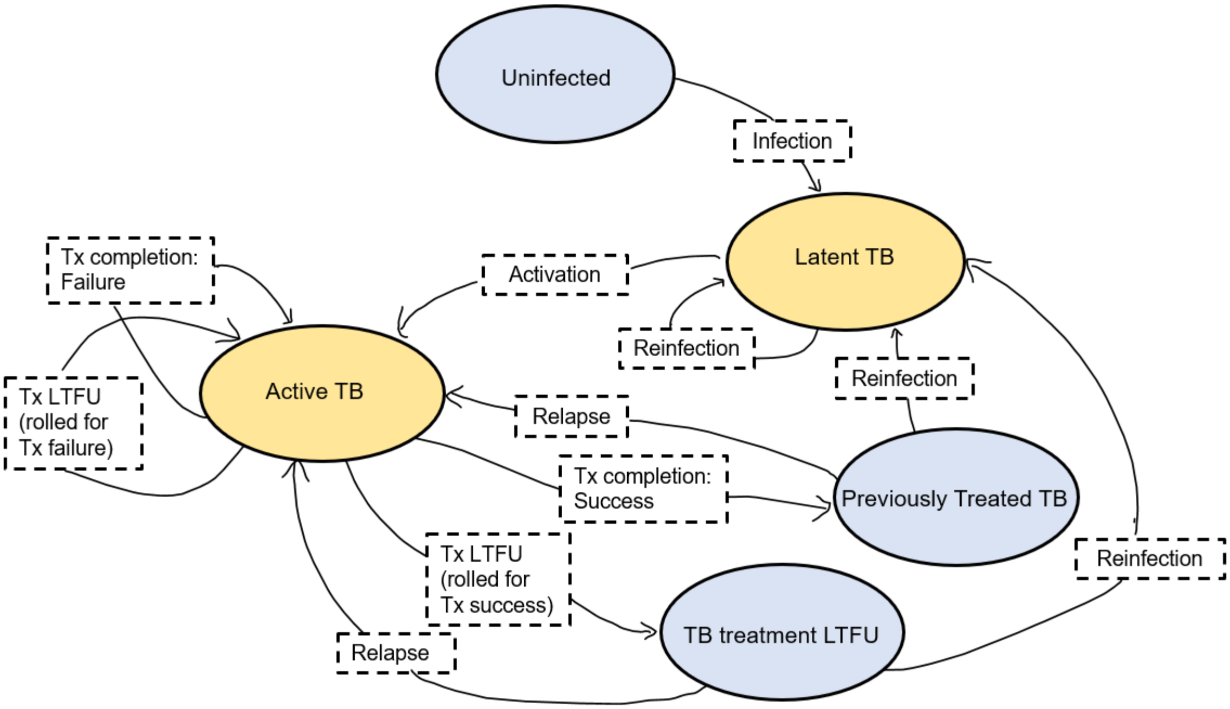

Figure S2. Overview of TB module health states and transitions.

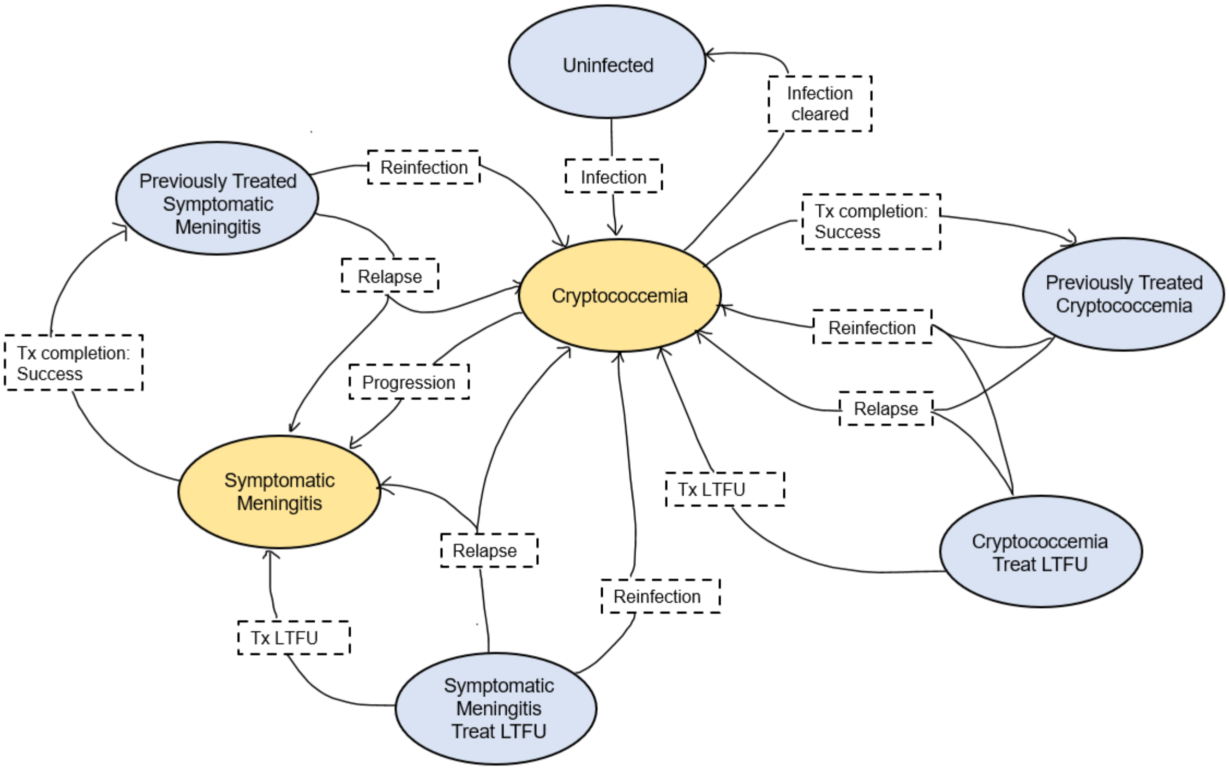

Figure S3. Overview of the cryptococcus module health states and transitions.

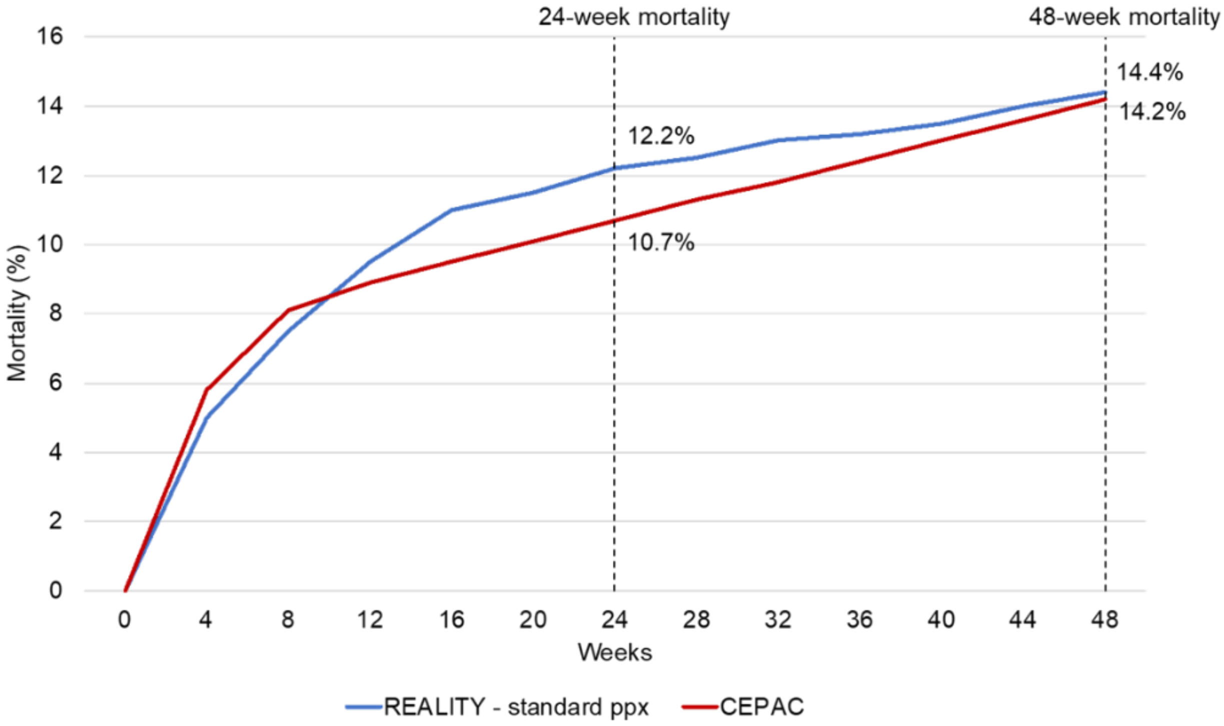

Figure S4. CEPAC-I projections of mortality compared with observed mortality in the REALITY trial.

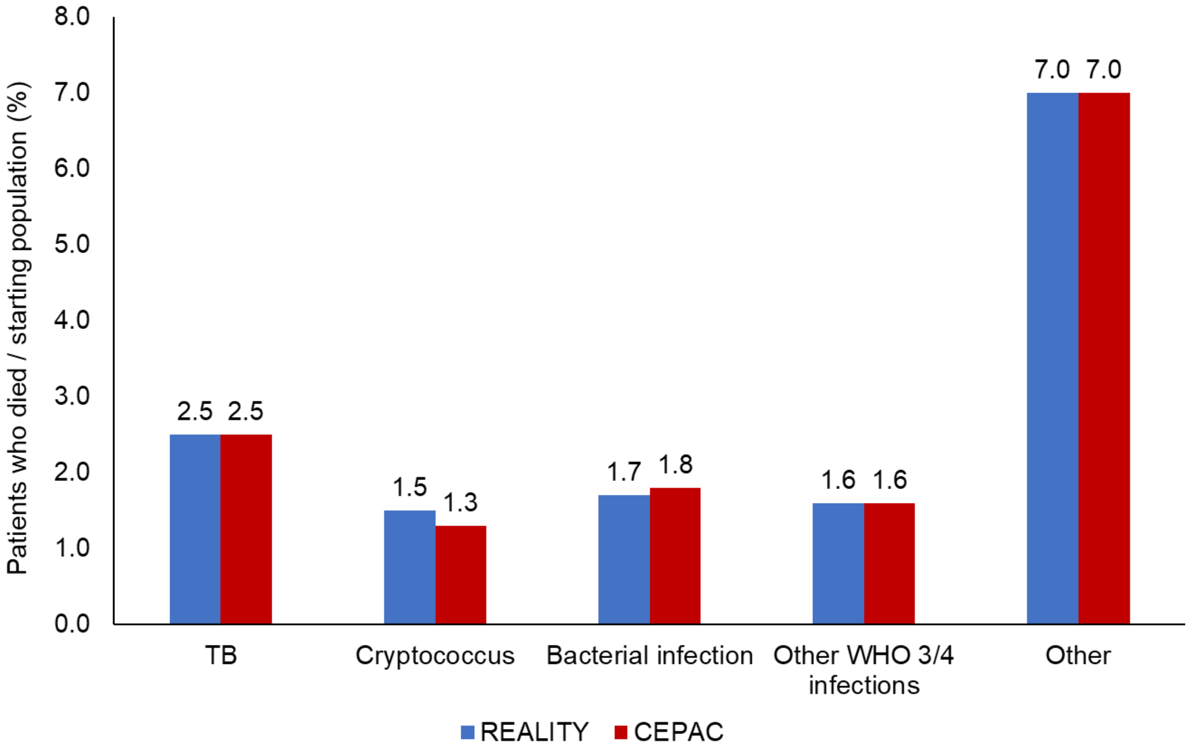

Figure S5. CEPAC-I projected cause of death compared with REALITY trial results for people initiating ART with AHD.

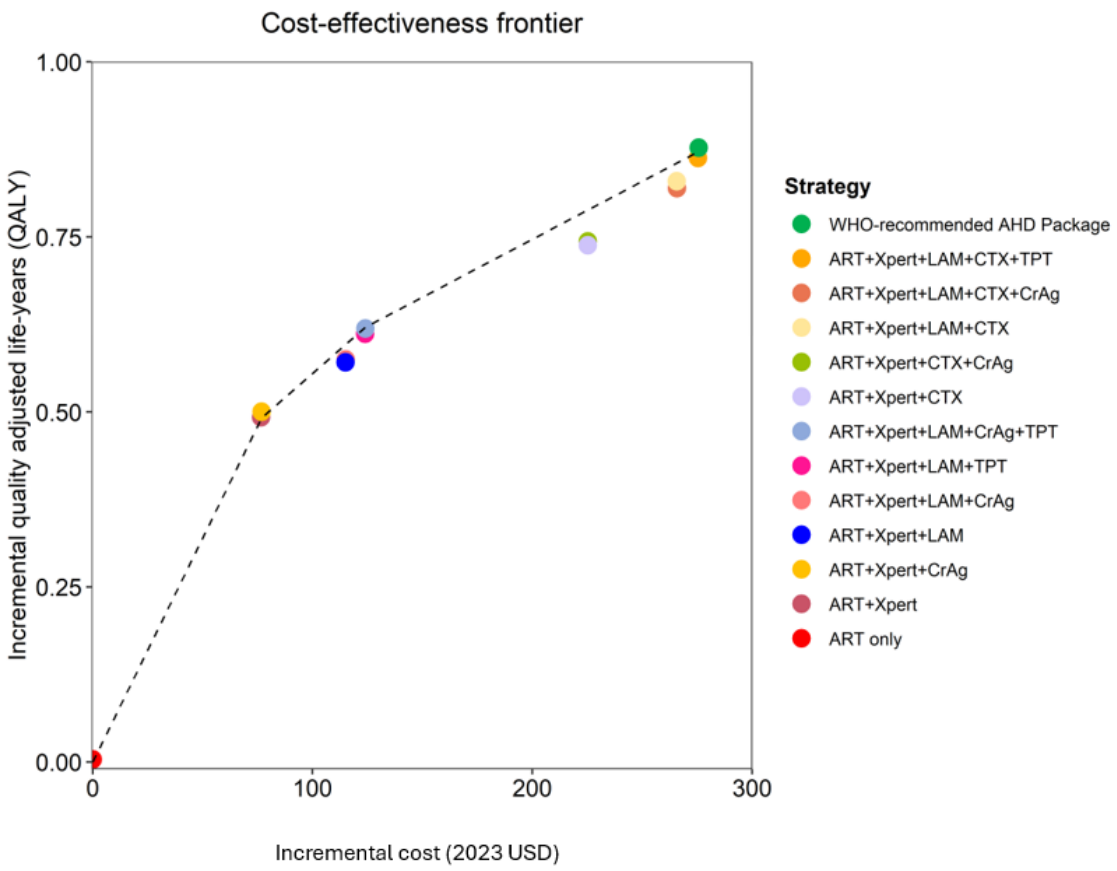

Figure S6. Model-projected incremental lifetime clinical outcomes and costs for all strategies with CD4 test available.

(A)

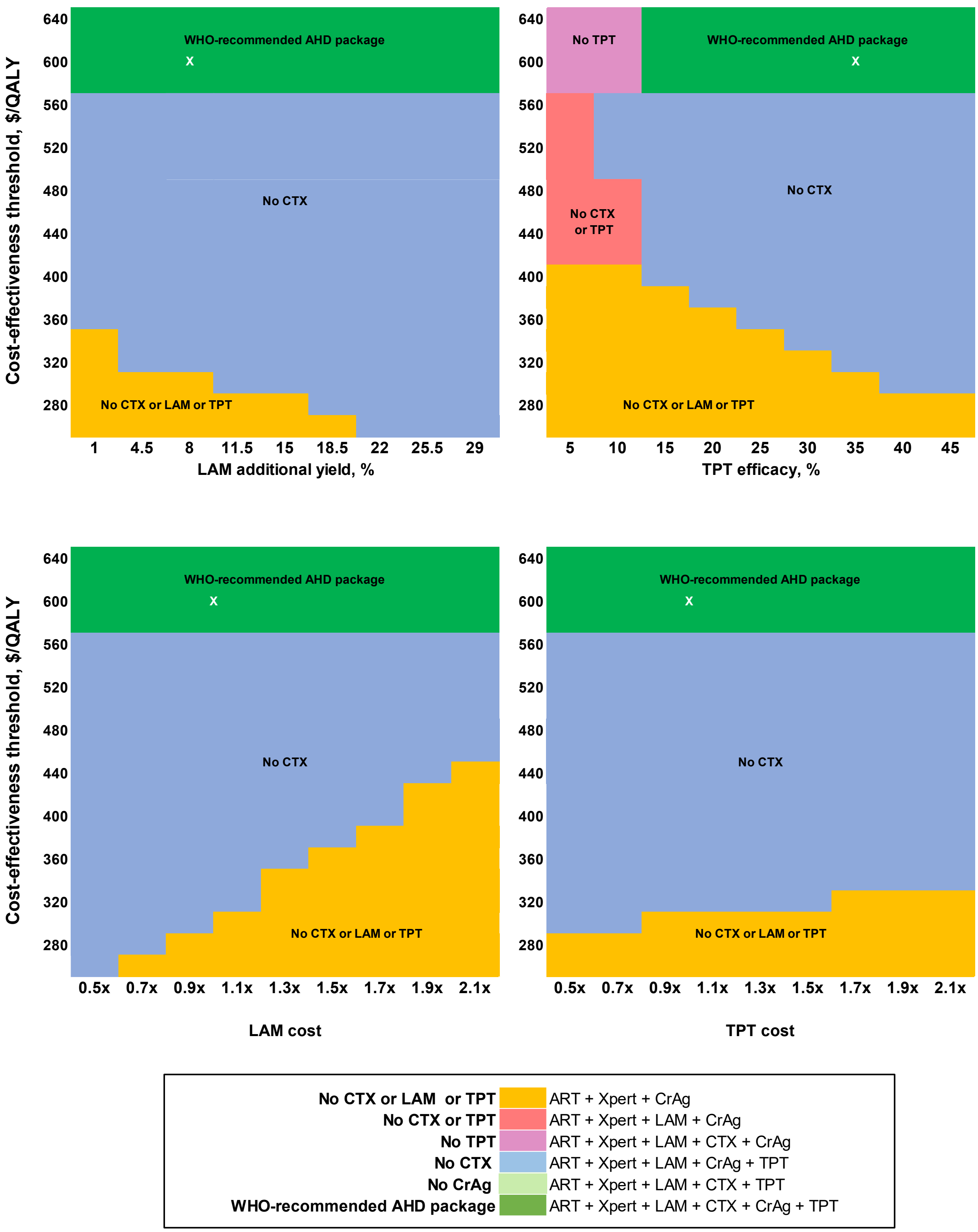
WHO-recommended AHD package
No CTX
No CTX or LAM or TPT
No TPT
No CTX or TPT
Cost-effectiveness threshold, $/QALY
LAM additional yield, %
TPT efficacy, %
LAM cost
TPT cost
640
600
560
520
480
440
400
360
320
280
1 4.5 8 11.5 15 18.5 22 25.5 29
5 10 15 20 25 30 35 40 45
0.5x 0.7x 0.9x 1.1x 1.3x 1.5x 1.7x 1.9x 2.1x
X
No CTX or LAM or TPT — ART + Xpert + CrAg
No CTX or TPT — ART + Xpert + LAM + CrAg
No TPT — ART + Xpert + LAM + CTX + CrAg
No CTX — ART + Xpert + LAM + CrAg + TPT
No CrAg — ART + Xpert + LAM + CTX + TPT
WHO-recommended AHD package — ART + Xpert + LAM + CTX + CrAg + TPT

(B)

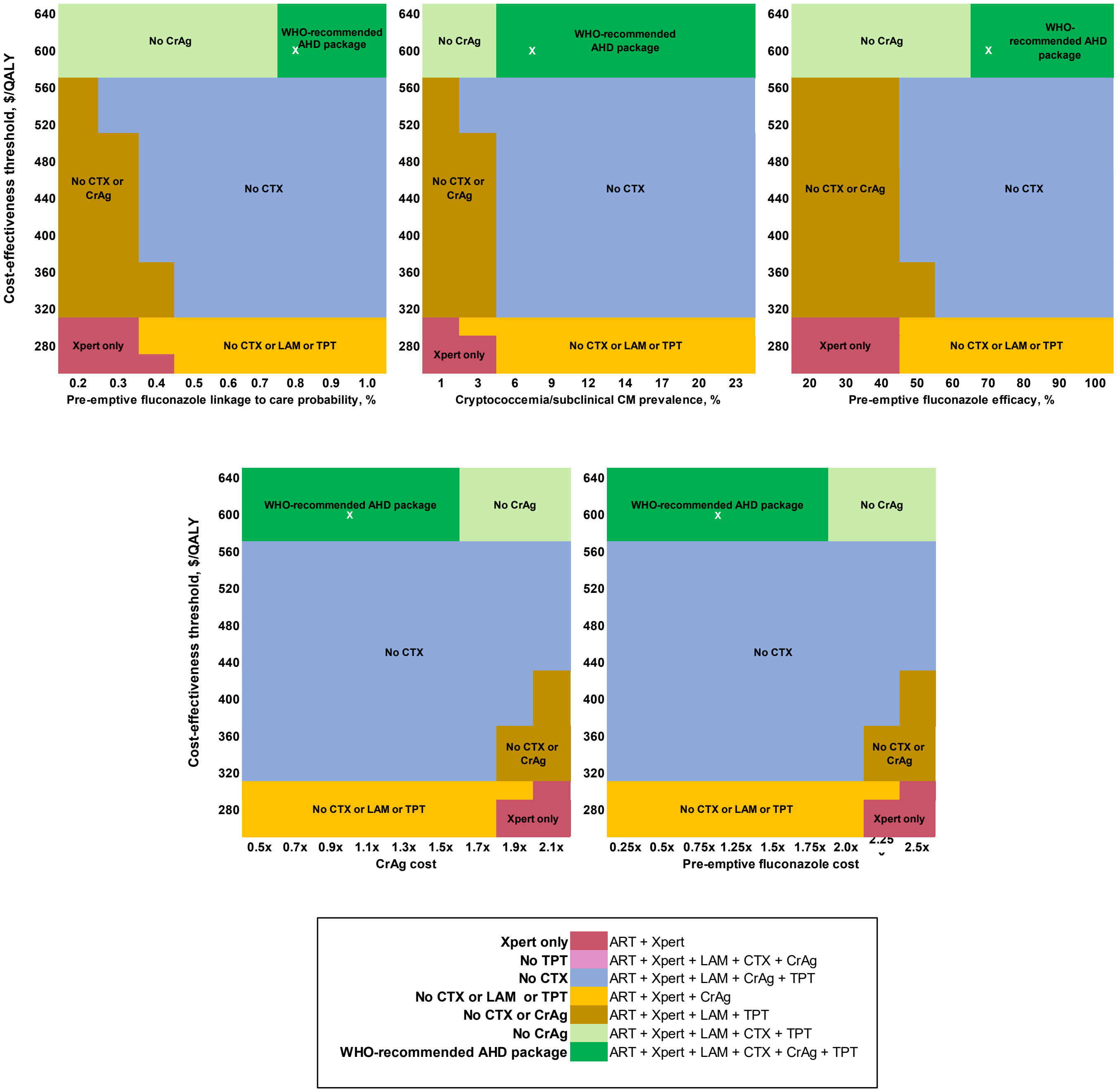
Cost-effectiveness threshold, $/QALY
640
600
560
520
480
440
400
360
320
280
No CrAg
WHO-recommended AHD package
x
No CTX or CrAg
No CTX
Xpert only
No CTX or LAM or TPT
0.2 0.3 0.4 0.5 0.6 0.7 0.8 0.9 1.0
Pre-emptive fluconazole linkage to care probability, %
1 3 6 9 12 14 17 20 23
Cryptococcemia/subclinical CM prevalence, %
20 30 40 50 60 70 80 90 100
Pre-emptive fluconazole efficacy, %
0.5x 0.7x 0.9x 1.1x 1.3x 1.5x 1.7x 1.9x 2.1x
CrAg cost
0.25x 0.5x 0.75x 1.25x 1.5x 1.75x 2.0x 2.25x 2.5x
Pre-emptive fluconazole cost
Xpert only ART + Xpert
No TPT ART + Xpert + LAM + CTX + CrAg
No CTX ART + Xpert + LAM + CrAg + TPT
No CTX or LAM or TPT ART + Xpert + CrAg
No CTX or CrAg ART + Xpert + LAM + TPT
No CrAg ART + Xpert + LAM + CTX + TPT
WHO-recommended AHD package ART + Xpert + LAM + CTX + CrAg + TPT

(C)

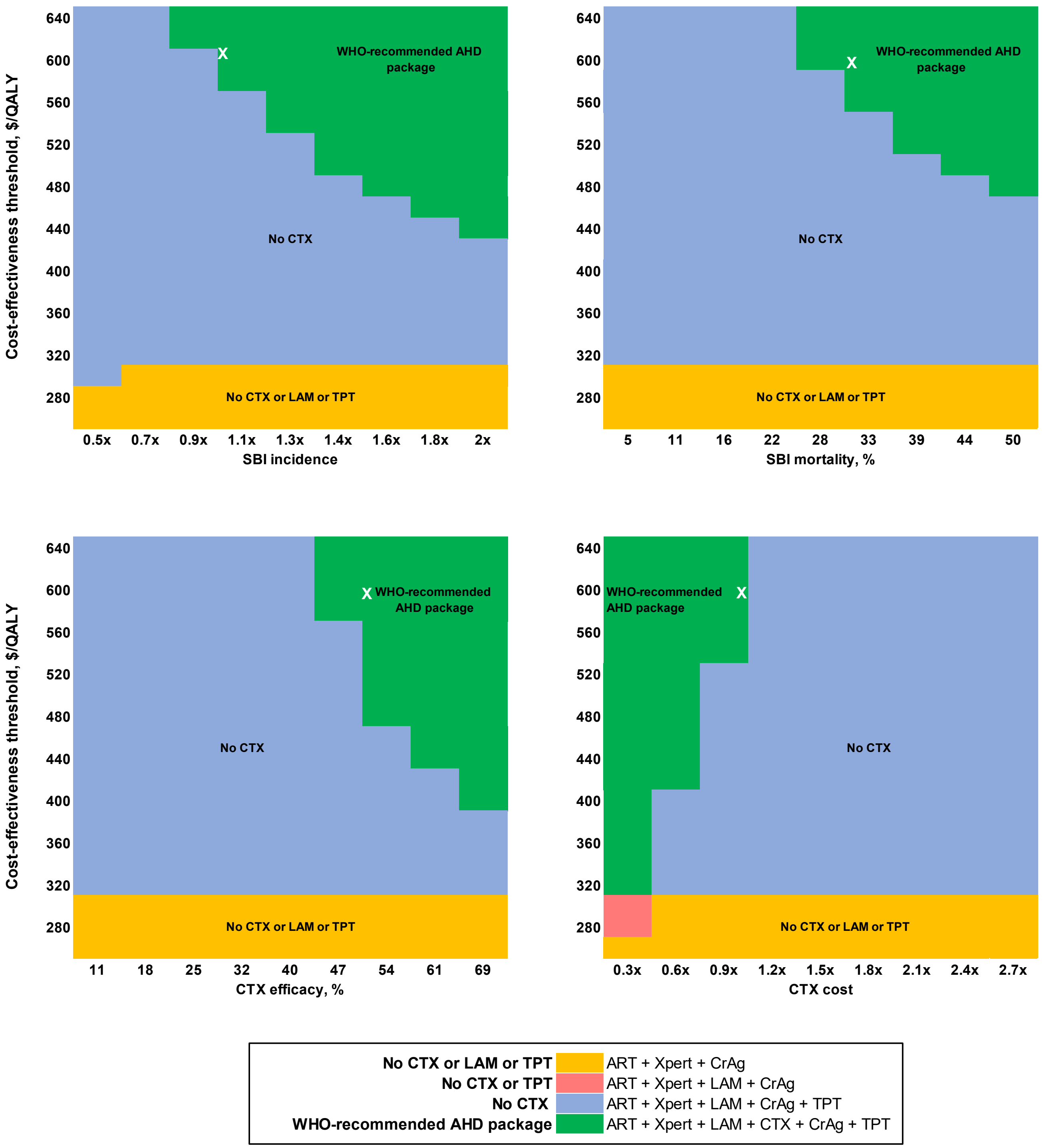

Figure S7. Univariate sensitivity analyses for selected clinical and cost parameters over a range of cost-effectiveness thresholds.

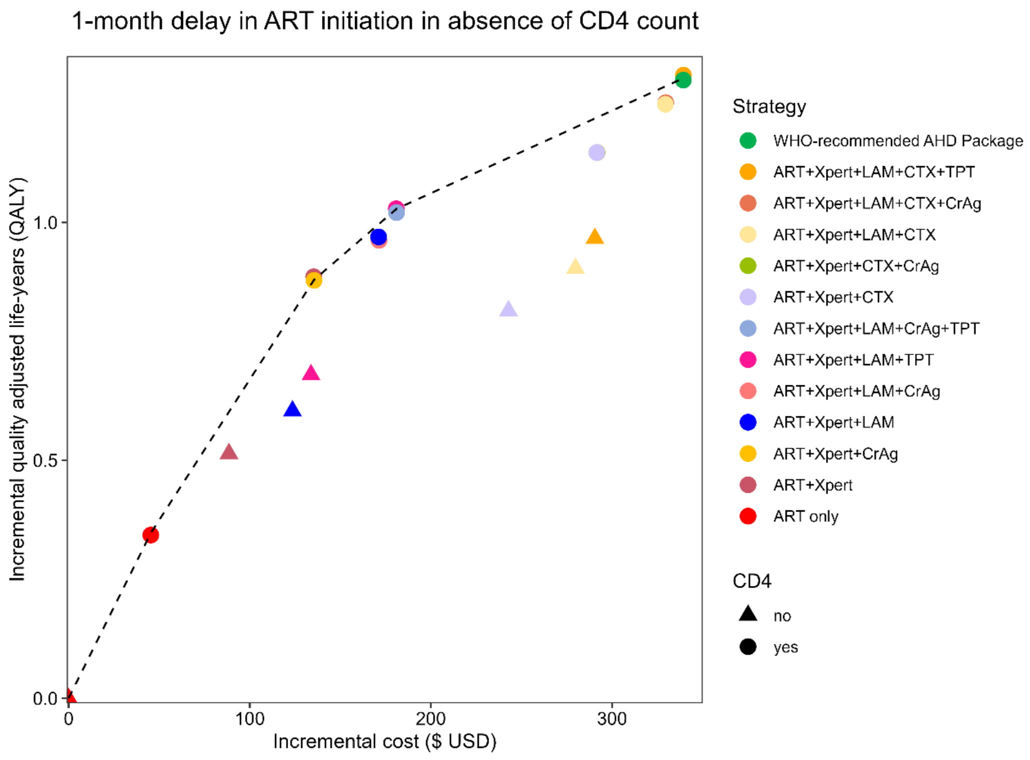

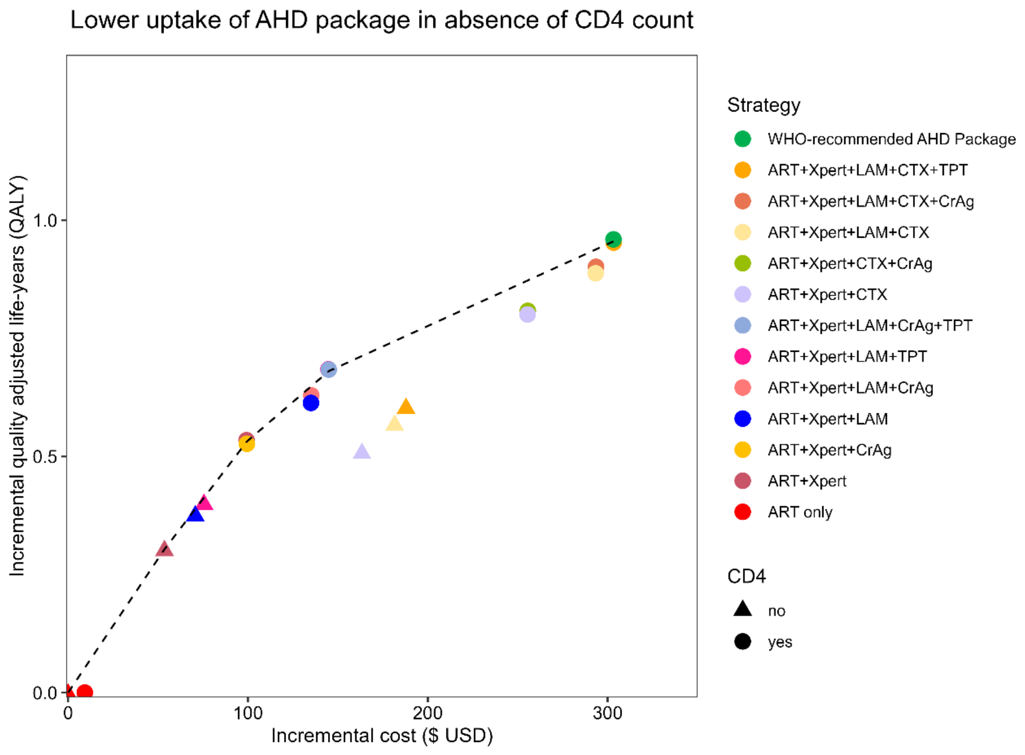

Figure S8. Model-projected incremental lifetime clinical outcomes and costs for all strategies with and without a CD4 test available in two scenarios.

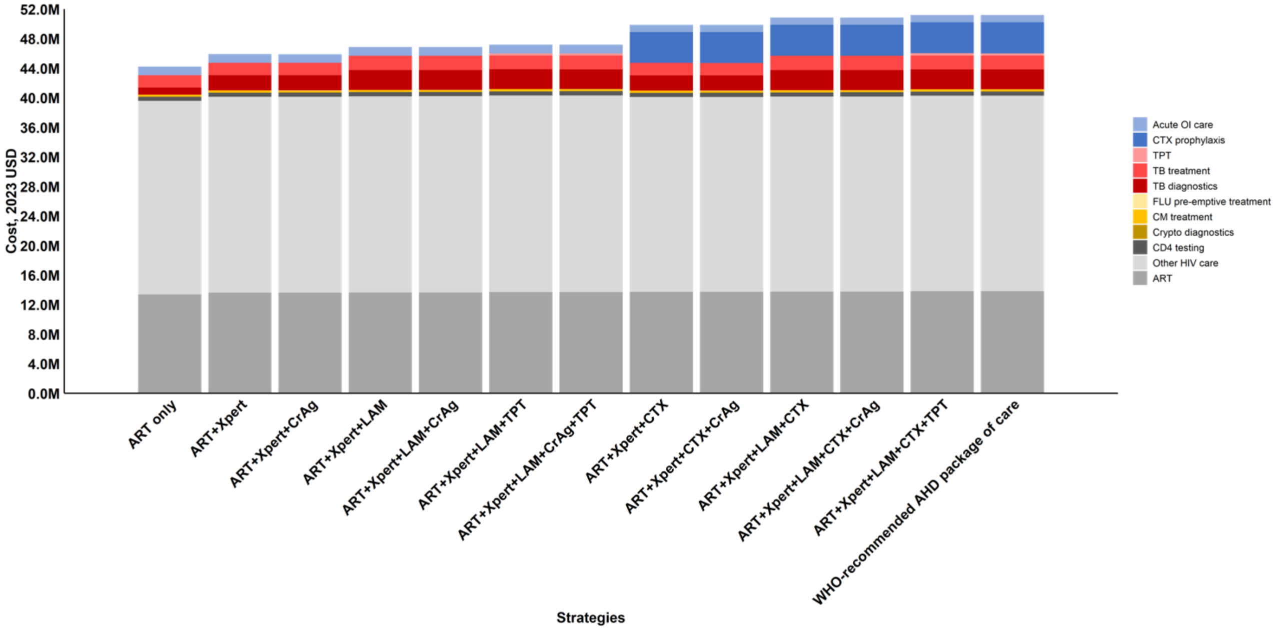

Figure S9. Detailed budget impact analysis for the *WHO-recommended AHD Package* for people living with HIV in Malawi.
